# Adaptive cognitive control in 4 to 7-year-old children and potential effects of school-based yoga-mindfulness interventions: an exploratory study in Italy

**DOI:** 10.3389/fpsyg.2025.1379241

**Published:** 2025-01-24

**Authors:** Lisa Toffoli, Giulia Stefanelli, Giulia Manca, Fiorella Del Popolo Cristaldi, Gian Marco Duma, Michele Guidi, Francesca Incagli, Luca Sbernini, Vincenza Tarantino, Giovanni Mento

**Affiliations:** ^1^NeuroDev lab, Department of General Psychology, University of Padova, Padova, Italy; ^2^IRCCS E. Medea Scientific Institute, Conegliano, Treviso, Italy; ^3^Cooperativa Progetto Insieme, Padova, Italy; ^4^IRCCS Carlo Besta Neurological Institute Foundation, Milano, Italy; ^5^Centro Regionale di Ricerca e Servizi Educativi per le Difficoltà di Apprendimento–Polo Apprendimento, Padova, Italy; ^6^Department of Psychology, Educational Science and Human Movement, University of Palermo, Palermo, Italy

**Keywords:** adaptive cognitive control, yoga-mindfulness, implicit learning, interference control, reactive and proactive control, motor preparation and inhibition, school, executive functions

## Abstract

**Introduction:**

Recent findings showed that adaptive cognitive control (CC) can be instantiated by bottom-up mechanisms, including statistical contingency of event occurrence. However, the developmental evidence in this domain remains limited.

**Methods:**

To address this gap, our study delves into the exploration of different mechanisms underlying adaptive CC in a substantial cohort of young children (211 participants aged between 4 and 7 years). We utilized the Dynamic Temporal Prediction (DTP) task and a modified version of the Flanker task to assess the effect of context predictability on motor preparation/inhibition and interference control, respectively. Furthermore, as part of an exploratory study designed to evaluate the feasibility of a school-based program in Italy, all children underwent a re-testing session after an 8-week intervention involving yoga-mindfulness.

**Results:**

Results suggested that young children can exploit global probabilistic changes to optimize motor preparation/ inhibition while counterbalancing fatigue effects. Moreover, they successfully modulate interference control as a function of environmental contingencies, displaying more optimal conflict resolution when proactive control is engaged. Finally, we observed a post-intervention increase of the capability to implicitly adapt motor preparation/inhibition and a boosting effect on the interference control functions.

**Discussion:**

Overall, these findings confirmed that adaptive CC is already present in preschool-aged children, extending these results to include 4-years-olds. Additionally, school-based yoga-mindfulness programs are feasible and might improve children’s capability to flexibly and proactively adapt to environmental requests promoting cognitive proficiency.

## 1 Introduction

Cognitive control (CC) can be considered as an individual repertoire of cognitive functions (i.e., inhibition, flexibility, working memory) that allows the implementation of adaptive behavior in everyday life (Diamond, 2013; Miyake et al., 2000). For instance, being able to inhibit inappropriate behaviors at school or easily adjusting attentional resources according to environmental demands, are key abilities for positive short-and long-term outcomes. Indeed, many studies suggest that CC abilities during the first years of life hold great predictive power for later quality of life in terms of personal health, relationships, academic and work achievements (Diamond, 2020).

### 1.1 Adaptive cognitive control

Recently, it has been suggested that CC can be adjusted as a function of bottom-up, environmental regularities even in the absence of awareness (Abrahamse et al., 2016; Braem and Egner, 2018; Braem et al., 2019; Del Popolo Cristaldi et al., 2023; Mento and Granziol, 2020; Mento et al., 2022). Put simply, CC can rely on bottom-up processes (i.e., associative and statistical learning) to extract environmental regularities to generate internal predictive models (Abrahamse et al., 2016). These latter allow an optimization of cognitive resources allocation, translating into more flexible and adaptive behaviors. This new perspective on CC aligns with the Dual Mechanism Control (DMC) model proposed by Braver et al. (2021), according to which CC adaptation depends on the flexible interplay between two mechanisms, namely proactive and reactive ones. In low-conflict prediction scenarios, reactive control operates as an energy-efficient mechanism, utilizing cognitive resources only when necessary. Conversely, in high-conflict prediction contexts, a proactive modality, while cognitively demanding, can offer a more efficient adaptive response. The adaptive interplay between reactive and proactive control mechanisms should result in a flexible adaptation toward environmental changing demands. Therefore, when transitioning from a low-conflict to a high-conflict environment, we might anticipate an improvement in cognitive conflict management or inhibitory control, driven by the increased reliance on proactive strategies in the more demanding context. Importantly, these two CC modalities have been widely investigated using tasks requiring explicit maintenance of cue-target relationships, like the AX-CPT (Gonthier et al., 2016). However, these tasks might be less appropriate to study adaptive CC in children, as limited working memory and/or metacognitive strategies might represent significant confounds (Chevalier et al., 2015; Gonthier et al., 2019). Consequently, tasks adopting implicit contextual changes (e.g., different levels of difficulties) represent a good alternative to study how appropriate reactive-proactive CC strategies are automatically instantiated in a bottom-up fashion.

### 1.2 Adaptive cognitive control development

Interestingly, developmental studies have shown that the transition from preschool to school age is a sensitive period not only for CC development (Diamond, 2013), but also for the refinement of its bottom-up adaptive modulation related to working memory and meta-cognitive development (Chevalier et al., 2020; Gonthier et al., 2019). Interestingly, behavioral and electrophysiological responses are modulated by the presence of conflict in preceding trials in adults and adolescents, but not children, suggesting greater reliance on reactive compared to proactive CC (Waxer and Morton, 2011). Additionally, children exhibit reduced engagement of neural areas, such as the anterior cingulate cortex and lateral prefrontal cortex, which are critical for moment-to-moment conflict management and adaptation (Wilk and Morton, 2012). More recent studies focusing on CC adaptation at the list or block level, provided evidence of a progressive transition from a preferential use of reactive to proactive control strategies from 5 to 10 years of age (Gonthier et al., 2019). However, children can engage in proactive CC from as early as 5 years old, provided the environment offers enough predictive scaffolding (Chevalier et al., 2015; Niebaum et al., 2021). In line with these results, in a series of studies from our lab, we showed that the refinement of adaptive CC is an age-sensible process (Del Popolo Cristaldi et al., 2023; Mento and Granziol, 2020), starting from preschool age and continuously developing through adolescence until adulthood. In these studies, we utilized a reaction time task called the Dynamic Temporal Prediction (DTP) task, which assesses CC adaptation in terms of motor preparation and inhibition. Specifically, Del Popolo Cristaldi et al. (2023) showed that the speed-accuracy trade-off adjustments as a function of contextual difficulty (i.e., higher or lower speed of target appearance) revealed that only adolescents, like adults, demonstrate early CC adaptation to global contextual changes. In contrast, children (ages 6–11) exhibit only late CC adaptation, suggesting it takes them longer to infer contextual regularities and adapt CC accordingly.

Conversely, other studies using list-wide proportion congruency manipulations have found that adaptive CC is early and stable from preschool age (Gonthier et al., 2021; Gonthier and Blaye, 2021). For instance, Gonthier et al. (2021) used a modified version of the Flanker task and found that children as young as 5 years, similar to adults, modulate cognitive interference as a function of contextual difficulty (e.g., reduced cognitive interference in blocks with high versus low proportions of incongruent trials). It is possible that in the absence of explicit requests (e.g., keeping in mind cue-target relationships) and with one instead of multiple cognitive mechanisms involved, CC adaptation is early and stable from at least preschool age. However, research on adaptive CC in children is limited, with methodologies and tasks varying significantly across studies. Therefore, it is crucial to confirm previous findings using similar tasks to better understand how adaptive CC develops and identify the variables that influence this process.

### 1.3 Yoga and mindfulness as potential trainings for enhancing adaptive cognitive control

Given the relevance of adaptive CC, it is crucial to understand the role of the environment in potentially boosting or hampering its development. To this regard, a bunch of studies have revealed that early environmental stressors (e.g., social stress, low socioeconomic status) can hamper CC development (Diamond and Ling, 2016; Miguel et al., 2023). Amongst them, the covid-19 pandemic, during which our study was conducted, represents an unprecedented global challenge for psychological wellbeing. Indeed, during the lockdown children were limited in a number of activities that are positively associated with CC development, such as play with peers, social interactions and physical activities (Fleer et al., 2020; Schmidt et al., 2015; Tvardovskaya et al., 2020; Veraksa et al., 2020). Fortunately, the environment can also boost CC development, as in the case of training or activities engaging psychophysical practices like yoga-mindfulness (Diamond and Lee, 2011; Diamond and Ling, 2016).

Yoga is an ancient practice that favors mental and physical well-being, by promoting mind–body harmonization through breathing and movement exercises (Kabat-Zinn, 1990). This practice is often combined with mindfulness, favoring conscious awareness and attention toward the present moment (Kabat-Zinn, 1994; Malinowski, 2013). By promoting focus on body sensations, emotions, mental images or mental dialogue, mindfulness results in a reduction of mind-wandering states or unadaptive behaviors (Creswell, 2017). Apart from the beneficial effects on physical fitness and emotional state, yoga practice improves several aspects of cognition and executive functions (Luu and Hall, 2016). In this regard, findings suggest that yoga-mindfulness interventions may be efficient in improving cognitive abilities such as attention, self-regulation, mental processes, school performance and behavior (Incagli et al., 2020; Lutz et al., 2008, 2009; Hagins and Rundle, 2016), eventually boosting adaptive CC development. Indeed, apart from the physical exercises, yoga also includes mental techniques that are supposed to drive CC benefits. This holds particular significance for children, as optimal CC enables the necessary behavioral self-control to adjust well to learning environments and is a reliable indicator of academic success in arithmetic and reading throughout the school years (Luu and Hall, 2016). It follows that the number of yoga mindfulness-based interventions aimed at enhancing developmental outcomes in children and adolescents has increased in the past years. However, the generally limited quality of the research has limited the inferences that can be drawn (Greenberg and Harris, 2011).

Telles et al. (2013) investigated the effects of a yoga intervention on children aged 8–13 after 3 months, finding substantial improvements in inhibitory control, as measured by the Stroop task (Stroop, 1935). Also, compared to physical activity Yoga was shown to positively impact executive function, as assessed by the Tower of London test (Manjunath and Telles, 2001). Similarly, Chaya et al. (2012) found that yoga was as effective as physical activity in enhancing cognitive and physical performance in over 200 children aged 7–9. It also showed to have other benefits such as improving general, parental and total self-esteem.

Tang et al. (2007) demonstrated that mindfulness-based interventions can significantly improve executive attention on the Attention Network Task (Fan et al., 2002) after just 5 days of training, highlighting the benefits of such practices for cognitive development in children and adolescents. However, research on younger preschool-aged children is limited, although it is a critical period for CC and self-control development (Diamond, 2013, 2020). Flook et al. (2010) examined an 8-week school-based mindfulness curriculum for second and third graders, which included breath awareness and movement activities. While no overall effects were observed on parent or teacher reports of cognitive control, significant improvements were noted in students with lower baseline levels of control.

Further supporting these findings, Tang et al. (2012) showed that mindfulness-based interventions in children aged 4–5 years led to improvements in inhibitory control and executive function, similar to effects observed in older age groups. This suggests that interventions promoting sustained attention to mind–body-environment sensations can enhance emotional regulation and cognitive control (Incagli et al., 2020).

Traditionally, yoga-mindfulness interventions were designed for clinical interventions (Segal et al., 2002). However, in recent years these practices have been integrated into the more natural settings (e.g., work or school environment). Many studies, with and without a control group, highlight an association with improved stress management and reduced negative emotions and thoughts, as well as with enhanced school performance, attention and CC (Bazzano et al., 2022; Frank et al., 2017; Good et al., 2016; Hart et al., 2022; Serwacki and Cook-Cottone, 2012; Sibinga et al., 2016). Given these considerations, there has been an increase in yoga-mindfulness school-based interventions (Serwacki and Cook-Cottone, 2012). However, the vast majority of studies assessing the feasibility and effectiveness of these interventions from preschool to adolescence was mainly conducted in the USA (Rawana et al., 2018), leaving this practice underrepresented in European countries such as Italy. To our knowledge, the only Italian example is the study by Crescentini et al. (2016), who implemented a school-based 8-week yoga-mindfulness intervention for primary school children (7–8 years old). Their 8-week program was predominantly mindfulness-oriented, with an intensive schedule (3 sessions per week) of increasing durations (45 min to 1.5 h). To assess the effectiveness of their intervention, the researchers utilized teachers’ rating scales for childrens’ problematic behaviors (Child Behavior Checklist (CBCL); Achenbach and Edelbrock, 1991; Frigerio et al., 2004; Conners’ Parent Rating Scales-Revised (CPRS-R); Conners, 2001; Conners et al., 1998; Nobile et al., 2007) and one self-evaluation scale for children (short mood and feeling questionnaire, Crescentini et al., 2016). The results suggested positive effects on both attention and internalizing problems, as observed in the teachers’ rating scales. Although promising, this study does not provide a comprehensive overview of the interventions’ effects, as it lacks both objective measures of children’s performance and parental perspective on children’s everyday behavior at home. Furthermore, it is essential to extend this kind of study to the preschool age, as the benefits of yoga-mindfulness may be greater for preschool-aged children compared to adolescents (Diamond and Lee, 2011). This might be related to the fact that the preschool period is pivotal for both emotional and cognitive control development. Hence, this period might be an optimal window to provide this kind of experience (Sun et al., 2021).

### 1.4 Rationale of the present study

The main goal of the present study is to confirm previous findings on adaptive CC during a sensitive window for its development (4–7 years of age). To this purpose, we recruited a large cohort of children (*N* = 211) and used two computerized tasks assessing different aspects of adaptive CC, the Dynamic Temporal Prediction task (DTP; Del Popolo Cristaldi et al., 2023; Mento and Granziol, 2020) and a modified version of the Flanker task (Gonthier et al., 2021; Gonthier and Blaye, 2021). As a secondary aim, this study provides an exploratory investigation of the feasibility of conducting a yoga-mindfulness school-based intervention (involving both kindergarten and primary school classrooms) in the Italian context. Moreover, it provides—for the first time—preliminary findings on its effect on adaptive CC.

The DTP task measures how children implicitly adapt CC (i.e., trade-off between motor preparation and inhibition) over time. Specifically, the task entails a manipulation of the stimulus onset asynchrony (SOA) distribution, leading to “fast” (i.e., 70% short 400 ms SOAs) and “slow” (i.e., 70% long 1,000 ms SOAs) blocks. These two types of blocks should elicit, respectively, a reactive and proactive control strategy, since in the “slow blocks” the hazard function induces higher motor preparation and, in turn, increased need for inhibitory control (Los et al., 2017). The blocks are presented in a fixed sequence (i.e., slow-1, fast-1, slow-2, fast-2) to study speed-accuracy trade-offs adjustments between the blocks. Previous studies (Del Popolo Cristaldi et al., 2023; Los et al., 2017; Mento and Granziol, 2020) found that efficient CC adaptation in this task is reflected by improved performance in “fast” compared to “slow blocks.” This adaptation is especially clear when focusing the analysis on short SOAs. Based on Del Popolo Cristaldi et al. (2023), we expected (H1a) that children of this age would demonstrate behavioral adaptation (i.e., speed-accuracy trade-off) only in the second part of the task (late adaptation) when considering short SOAs. This would be reflected as stable or improved performance from slow-2 to fast-2. Moreover, we expected an optimization of this late adaptation in the post-yoga session compared to the pre-yoga session (H1b). Importantly, the DTP task differs from classical task measuring CC adaptation as it does not include explicit conflict. While prior research has predominantly emphasized conflict as the primary trigger for shifts between reactive and proactive control modes (e.g., Botvinick et al., 2001; Braver, 2012; Kerns et al., 2004), CC adaptations can also arise from the predictability of cognitive demands, even in the absence of conflict (Munakata et al., 2023). Other lines of research further corroborate the idea that CC adaptation can occur in the absence of cognitive conflict. For instance, studies focusing on response inhibition (Doekemeijer et al., 2023; Giesen and Rothermund, 2014; Van Gaal et al., 2009) show that item-specific manipulations can induce automatic adaptations of inhibitory control (Logan and Cowan, 1984; Logan, 1988; Shiffrin and Schneider, 1977).

Our modified Flanker task measures how children implicitly adapt CC (i.e., interference control) in different predictive contexts. Specifically, the task entails a list-wide proportion congruency manipulation to create a non-predictive (i.e., 50% congruent trials) and a predictive (i.e., 75% congruent trials) block. The blocks are designed to elicit greater reliance on, respectively, proactive and reactive control strategies. The non-predictive block requires increased control to manage cognitive interference. Transitioning to the second predictive block (mostly congruent) introduces a higher proportion of congruent trials, creating an environment predictive of lower conflict demands. As a result, we expected greater reliance on reactive control in responding to incongruent trials within this block. This shift should result in an overall increase in cognitive interference in the predictive compared to the non-predictive block. Therefore, efficient CC adaptation in this task is reflected by reduced congruency effect (speed-accuracy trade-offs) in the non-predictive compared to the predictive block. Based on Gonthier et al. (2021) we expect (H2a) a smaller congruency effect (speed accuracy trade-off) in the non-predictive compared to the predictive block. Moreover, we expect that the degree of this modulation will increase in the post-yoga session, compared to the pre-yoga session (H2b).

Importantly, the school-based yoga-mindfulness intervention might have a more pronounced effect on the DTP task compared to the Flanker task. This is because previous studies indicate that the DTP task, which requires both motor preparation and inhibitory control, follows a prolonged developmental trajectory for adaptive CC (Del Popolo Cristaldi et al., 2023). In contrast, the Flanker task, associated with early and stable developmental patterns of adaptive CC (Gonthier et al., 2021), may offer less potential for improvement at this developmental stage.

Finally, we evaluated the impact of the yoga-mindfulness intervention on children’s daily behaviors and parental stress through parental questionnaires, specifically the Conners’ Parent Rating Scales - Revised (CPRS-R; Conners, 2001; Conners et al., 1998; Italian adaptation by Nobile et al., 2007) and the Parenting Stress Index (PSI; Abidin, 1995; Italian adaptation by Guarino et al., 2008). Consistent with previous research (Crescentini et al., 2016), we anticipated a general reduction in behavioral problems and parental stress as a result of the intervention (H3). Indeed, mindfulness-based interventions have been shown to improve cognitive control, attention and stress management (Bazzano et al., 2022; Frank et al., 2017; Good et al., 2016; Hart et al., 2022; Serwacki and Cook-Cottone, 2012; Sibinga et al., 2016). It is crucial to mention that the intervention was carried out during the covid-19 pandemic, when many restrictions were still in place in Italy (October 2021–March 2022). This challenging situation poses a limitation to our second aim as (1) we were unable to recruit a control group during this period, (2) we had to postpone the post-intervention session (initially scheduled for January 2022). Notably, the main focus of the yoga-mindfulness intervention was to assess the practical feasibility of this activity as a school-based program in the Italian context. Therefore, its effects on adaptive CC must be considered as exploratory.

## 2 Methods

### 2.1 Ethic statement

Children’s parents provided written consent for their children’s participation. All experimental procedures were approved by the Ethics Committee of the School of Psychology of the University of Padua (protocol no. 3666) and were conducted according to the principles expressed in the Declaration of Helsinki.

### 2.2 Participants

A total of 211 participants were initially enrolled. Children (4–7 years old) were recruited from the “Istituto Comprensivo G. Santini” school in Noventa Padovana, in the Venetian Region of Italy. Non-verbal intelligence was assessed using the Colored Progressive Matrices (CPM; Raven and Court, 1938). The following exclusion criteria were applied: CPM score of 2 standard deviations below the population mean (*N* = 5), certified sensory, neurological or psychiatric disorders (*N* = 3), difficulties in task execution (*N* = 5) and scarce Italian comprehension (*N* = 2). Moreover, 22 children were excluded due to technical issues. Finally, children completed two computerized experimental tasks (DTP and Flanker task). Global accuracy scores were calculated separately for each task, and participants with a score below 65% were excluded from the analysis of the respective task (*N* = 0 for the DTP task and *N* = 45 for the Flanker task). The final sample comprised 174 children (82 F; Mean age = 6 years, SD = 0.8; see Supplementary Figure S1) and 138 children for the Flanker task (67 F; Mean age = 6 years, SD = 0.8; see Supplementary Figure S2).

### 2.3 Materials

#### 2.3.1 The Dynamic Temporal Prediction task

The Dynamic Temporal Prediction task (Del Popolo Cristaldi et al., 2023; Mento and Granziol, 2020) is a speeded target detection task that allows the assessment of adaptive cognitive control based on both local (trial-by-trial hazard expectation) and global (block-by-block expectation) temporal contingencies. Both these sources of predictability can induce temporal expectancy allowing an implicit adaptive balancing between proactive and reactive action control. Indeed, the contingency manipulation in the DTP task is fully uninstructed, preventing reliance on declarative probabilistic knowledge that might support efficient performance through compensatory mechanisms. Importantly, unlike traditional cognitive control paradigms, such as the Stroop, Flanker or Simon tasks, the DTP task does not include a conflict condition. This eliminates any potential confound arising from excessive working memory load or complex instructions, as the only instruction is to press a button when the target occurs. Here we used the same DTP task as in Del Popolo Cristaldi et al. (2023).

##### 2.3.1.1 Trial structure

Each trial started with a warning visual stimulus (S1; picture of black camera lens surrounded by a circle), followed by the display of an imperative visual stimulus (S2; picture of a cartoon animal displayed at the center of the camera lens) that remained on the screen for a maximum of 1,500 ms. The inter-trial-interval (ITI) was randomly manipulated between 200 and 400 ms. Participants were required to press the spacebar of the keyboard with the index finger of their dominant hand as quickly as possible at target occurrence. To motivate children and encourage good performance, they were given the following instruction: “*Hi! These cute little animals are playing hide and seek in the woods! Your job is to take a photo of them as quickly as possible when they appear in view of your camera. You can take a photo by pressing the spacebar. Find them all! But take care—if you press the bar too soon or too late, they will run away!*.”

##### 2.3.1.2 List-wide manipulation

**Figure 1:**
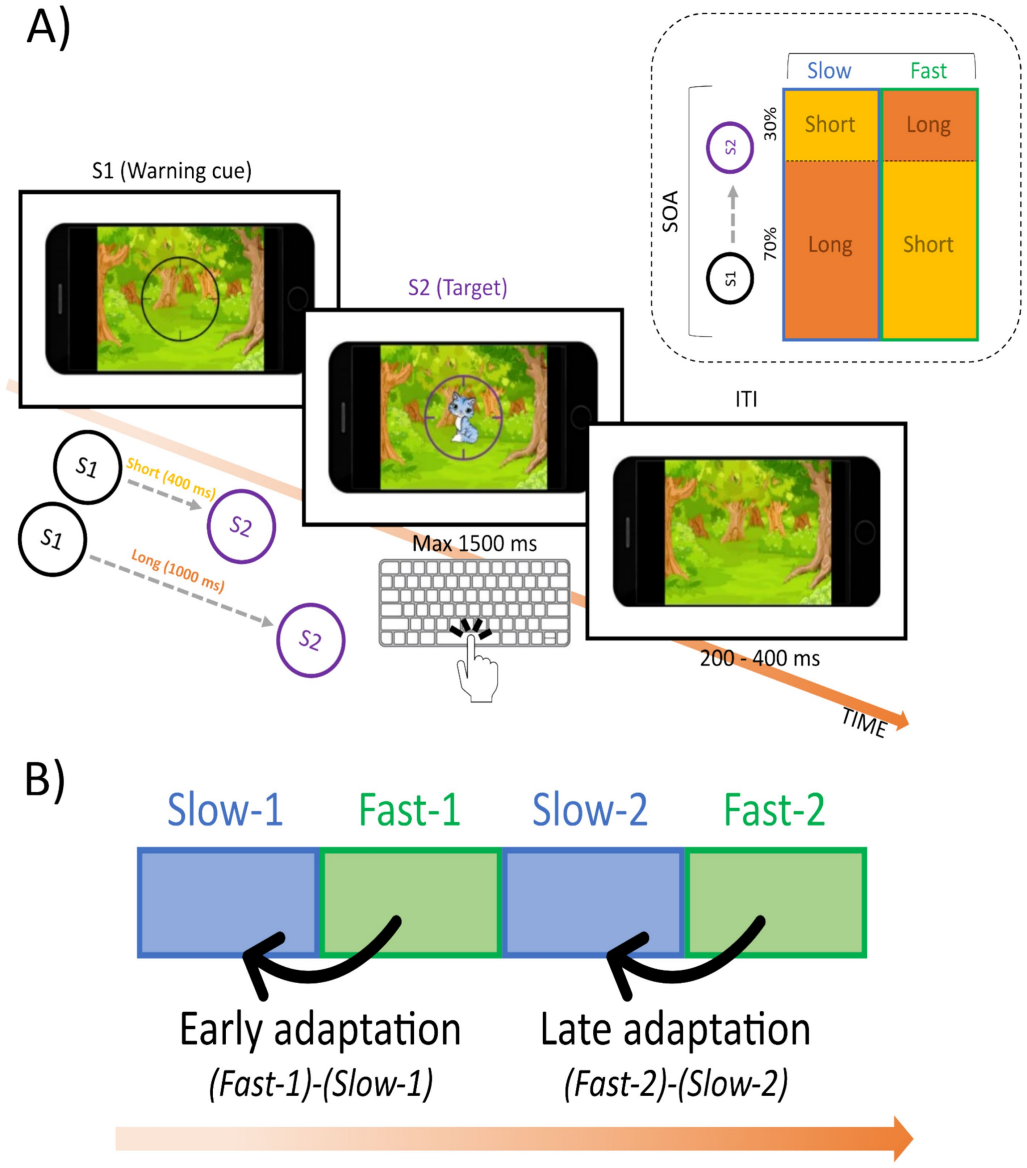
The Dynamic Temporal Prediction (DTP) task. **(A)** The task depicted a smartphone containing the task as subsequently described. The circle (S1) warned children on the presentation of the imperative S2 stimulus (an animal cartoon). Participants had to press the space button on the keyboard as quickly as possible at S2 onset. **(B)** Adaptive motor control and inhibition reflecting the progressive block-by-block performance adaptation was assessed creating a fixed block sequence (slow-1, fast-1, slow-2, fast-2) to assess early and late task adaptation.

The S1-S2 stimulus onset asynchrony (SOA) was manipulated so that two possible fixed foreperiod intervals were defined: short (400 ms) and long (1,000 ms). These two foreperiods reliably induce hazard-related, local prediction effects on reaction times (RTs) in children. Specifically, this manipulation is expected to bias the subjective temporal expectancy (Luce, 1986; Karlin, 1959; Woodrow, 1914) with faster detection of targets occurring at the longer SOA and slower at those appearing at the short SOA (Niemi and Näätänen, 1981). Indeed, as the temporal interval preceding a target increases, motor preparation to respond also intensifies. The global predictive context was established by manipulating different foreperiod distributions block-by-block using a list-wise logic. Namely, “fast blocks” included an *a priori* biased distribution toward the short SOA (70% short vs. 30% long SOA), known as non-aging distribution (Trillenberg et al., 2000); conversely, “slow blocks” included an a priori biased distribution toward the long SOA (30% short vs. 70% long SOA), known as aging distribution (see Figure 1A). Crucially, “fast blocks” are expected to elicit faster responses than “slow blocks”, reflecting an adjustment to the speed demands of the block.

The aim of the task was to investigate children’s ability to implicitly grasp contextual information (i.e., temporal contingencies) and use it to optimize behavior dynamically. Therefore, we manipulated the global predictive context delivering the two types of experimental blocks (“fast” and “slow”) in a fixed order between participants (i.e., slow-fast-slow-fast), for a total of four blocks. This order allowed to disentangle adaptation (reduced RTs in the last “fast block”) and fatigue (increase RTs in the last “fast block”), two aspects that would have been confounded in a fast-slow-fast-slow sequence (i.e., both adaptation and fatigue would be reflected as an increase in RTs in the last “slow block”). We expect adaptation to be reflected by performance mirroring this task structure: given the same accuracy, speed up of reaction times from the first (slow-1) to the second (fast-1) block (early adaptation), and from the third (slow-2) to the fourth (fast-2) block (late adaptation) (see Figure 1B). The task comprised a total of 160 trials (40 trials per block: “slow blocks” = 28 long SOAs & 12 short SOAs, “fast blocks” = 28 short SOAs & 12 long SOAs), for a total length of about 10 min. To ensure that behavioral adaptation occurred implicitly, participants were not informed about the list-wide manipulation and no pauses were introduced between blocks. All blocks were matched for sensorimotor requirements. Before starting the experimental session, participants performed a practice block of 10 trials to assess task instructions understanding. During practice participants received trial-by-trial feedback based on their performance (i.e., puzzled face for wrong, anticipatory [response before target appearance] or premature [<150 ms after target appearance] responses, sleepy face for correct but delayed [>800 ms after target appearance] responses, smiley face for correct and fast responses). No feedback was delivered during the experimental session.

#### 2.3.2 The Flanker task

This modified version of the Flanker (Gonthier et al., 2021; Gonthier and Blaye, 2021) allows the assessment of adaptive CC based on different proportions of congruent vs. incongruent trials along the task. These latter can induce prediction, allowing an implicit adaptive balancing between proactive and reactive cognitive control. Importantly, differently from the DTP, the Flanker task implies an explicit adaptation of interference control to different predictive contexts (i.e., blocks) across the task.

##### 2.3.2.1 Trial structure

**Figure 2:**
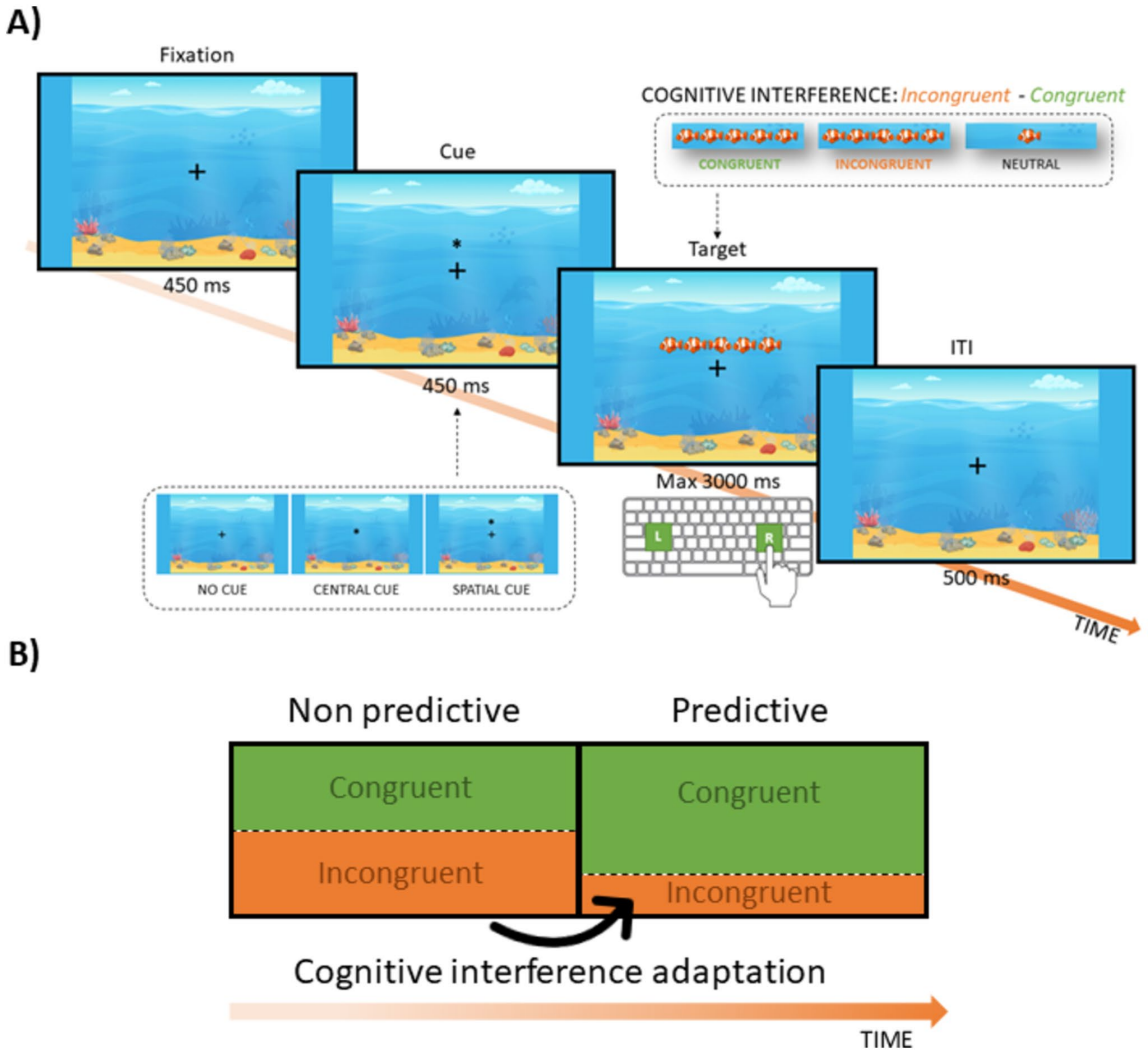
Modified version of the Flanker task. **(A)** The trial comprised a fixation cross, followed by a visual cue (i.e., no cue, central or spatial). Afterwards, the central cartoon fish (target) appeared in the middle of a horizontal string with other cartoon fish (flankers). Flankers’ compared to the target ‘s direction was either congruent or incongruent. In neutral conditions, no flanker was present. **(B)** List-wide manipulation. The percentage of congruent trials was manipulated list-wise creating a non-predictive (50% congruent trials) and a predictive (75% congruent trials) block. Adaptation of cognitive interference resolution to different predictive contexts was assessed creating a fixed block sequence (non-predictive-predictive).

Each trial began with a fixation cross (+) displayed for 450 ms in the center of the screen, followed by a warning visual stimulus (S1) for 450 ms and the target visual stimulus (S2) that stayed on the screen for a maximum of 3,000 ms. The warning visual stimulus (S1) consisted of an asterisk (✳) that either appeared in the same or opposite position of the target (spatial warning), at the center of the screen (central warning) or did not appear at all (no warning; the fixation cross was displayed instead). S2 consisted of the cartoon of an orange fish that could appear alone (neutral condition) or surrounded by flankers (identical orange fishes, two on the left and two on the right of the central target). In this latter case, the central fish could point toward the same direction of the flankers (i.e., congruent condition) or in the opposite direction of the flankers (i.e., incongruent condition). Moreover, S2 could appear above (50% of trials) or below (50% of trials) the fixation cross. The inter-trial-interval (ITI) was fixed (500 ms). Participants were required to press the “A” or “L” button on the keyboard according to the central fish direction (i.e., “A” if the fish was pointing left, “L” if the fish was pointing right) using the index finger of the left (for “A” button) and right (for the “L” button) hands (Figure 2A). Response keys were signaled by colored stickers. Children were prompted to respond as quickly and accurately as possible. To encourage good performance, children were given the following instructions: *“Nick is organizing a birthday party for his sister Maty! To surprise her, he wants to give her the beautiful fish Nemo! Would you like to help Nick in this adventure?”*

##### 2.3.2.2 List-wide manipulation

The aim of the task was to investigate children’s ability to implicitly grasp contextual information and use it to optimize behavior in a low motivational context. To this end, the proportion of congruent and incongruent trials was manipulated as follows: 50:50 (congruent:incongruent) in the first half of the task (non-predictive block) and 75:25 (congruent:incongruent) in the second half of the task (predictive block). This manipulation is supposed to bias children expectancy toward the congruent condition in the predictive block, resulting in a performance facilitation in congruent trials. In turn, this should be reflected in an increased cognitive interference among the predictive compared to the non-predictive block, as suggested by previous findings (Gonthier et al., 2021). Specifically, we expect adaptation to be reflected by performance showing reduced congruency effect (incongruent - congruent) in the non-predictive compared to the predictive block (Figure 2B). Importantly, the proportion of the four warning conditions (spatial, central, none) did not change across the whole task.

Two experimental blocks were delivered in a fixed order (i.e., non-predictive—predictive). Each block included 36 trials (non-predictive block: 16 congruent, [8 spatial cue, 4 central cue, 4 no cue], 16 incongruent, [8 spatial cue, 4 central cue, 4 no cue], 2 neutral; predictive block: 24 congruent, [12 spatial cue, 6 central cue, 6 no cue], 8 incongruent, 4 [4 spatial cue, 2 central cue, 2 no cue], 4 neutral), for a total of 72 trials. Importantly, neutral trials are considered catch trials and were not entered in the statistical analyses. The total length of the experiment was about 10 min. Participants were not told about the list-wide proportion congruency manipulation (i.e., different proportions of congruent and incongruent trials along the task). Moreover, no pauses were introduced between the two blocks. In this way, the changes between the blocks were never implicitly suggested by task interruptions. Instead, short pauses were provided every 18 trials, for a total of 4 pauses along the task to avoid fatigue in children. Both blocks were matched for sensorimotor requirements, as the visual stimuli and the required response were always the same across the experiment. The only differences were related to the changes in the predictive context experienced through the task (namely, the list-wide proportion congruency manipulation). Before starting the experimental session, participants were presented with a block of 12 practice trials to ensure they understood task instructions. During practice each participant received trial-by-trial feedback based on their performance. Specifically, a low auditory tone and a puzzled face in cases in which anticipatory (before target onset), premature (<150 ms after target onset) or wrong responses were provided; whereas a high auditory tone and a happy face were provided for correct responses. No feedback was delivered during the experimental session.

#### 2.3.3 Questionnaires

Initially, parents of children participating in this study were enlisted and requested to complete two questionnaires, the Conners’ Parents Rating Scales - Revised (CPRS-R; Conners, 2001; Conners et al., 1998; Italian adaptation by Nobile et al., 2007) and the Parenting Stress Index (PSI; Abidin, 1995, Italian adaptation by Guarino et al., 2008), both during pre-yoga and post-yoga sessions using the online survey platform Qualtrics (Qualtrics, 2019). In total, 234 questionnaires were collected but only 62 questionnaires were fully completed during both evaluation sessions. The Conners’ Parents Rating Scales - Revised (CPRS-R) is a parent questionnaire used in clinical and research settings to investigate children’s (3–17 years of age) behavior over the past month through statements describing children’s reactions in different contexts of daily life. The PSI is the most extensively used measure of parenting stress and specifically identifies “negative” stress in parents, the quality of the parent–child interaction, and the child’s individual characteristics. Only total scales of the CPRS-R (i.e., CGI-Total) and PSI (i.e., PSI total scale) questionnaires entered the analysis.

### 2.4 Experimental procedure

**Figure 3:**
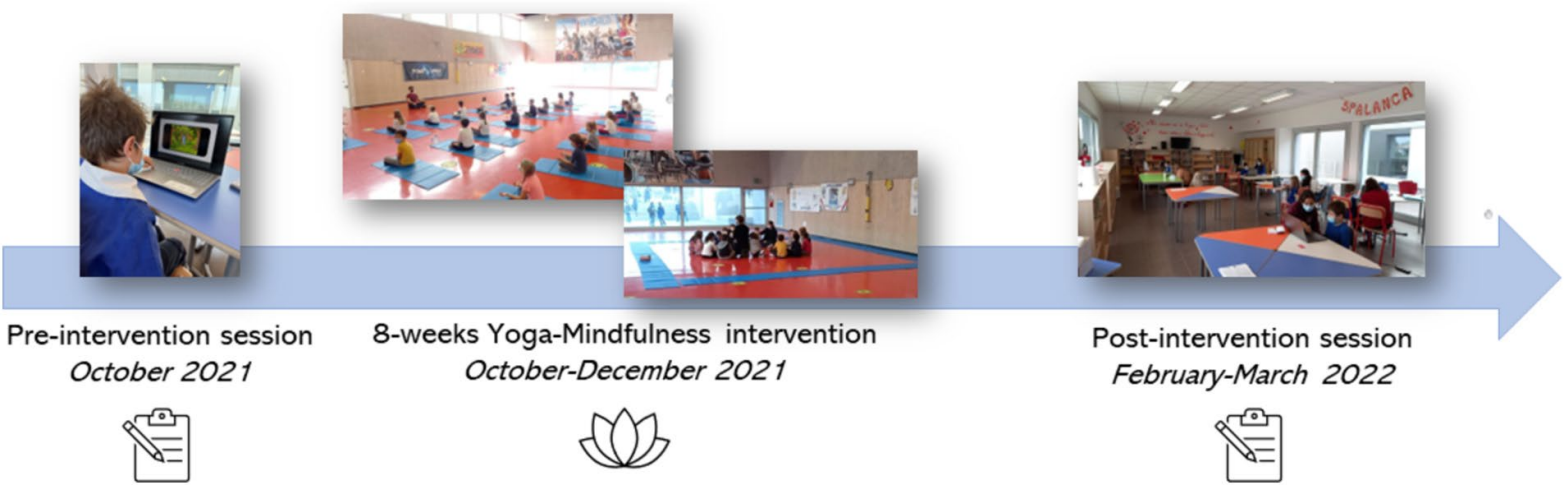
Yoga-mindfulness research intervention protocol timeline. The first evaluation session took place in October 2021 (Pre-intervention session), the intervention took place from October to December 2021 (Yoga-mindfulness intervention), lastly the second and final evaluation session took place in February–March 2022 (Post-intervention session).

This study is part of a larger project exploring the feasibility of school-based yoga-mindfulness interventions in Italian schools and their impact on cognitive control and emotional well-being in children and their families. Specifically, this study focused on the potential effects of the intervention on adaptive CC, leading us to select tasks that specifically target this aspect. The experimental procedure consisted of three main parts: (1) the pre-intervention assessment (October, 2021), (2) the intervention (October–December, 2021) and (3) the post-intervention assessment (February–March, 2022; see Figure 3). Both the pre-and post-intervention assessments included direct measures of childrens’ adaptive CC (i.e., DTP and Flanker tasks) and indirect measures of children’s behavior and emotional well-being (i.e., parental questionnaires: CBCL; Achenbach and Edelbrock, 1991; Frigerio et al., 2004; CPRS-R; Conners, 2001; Conners et al., 1998; Nobile et al., 2007). Moreover, we assessed parental stress (PSI; Abidin, 1995). Children’s assessments took place at their school, “Istituto Comprensivo G. Santini,” Noventa Padovana, (Padova, Italy). The testing occurred in a comfortable and quiet room (i.e., the library), with children tested in small groups for organizational reasons (each child completed the task individually and was supervised by an experimenter). The DTP and Flanker tasks were created and delivered using the OpenSesame software (Mathôt et al., 2012). Questionnaires were administered through the online platform Qualtrics (Qualtrics, 2019), allowing parents to conveniently complete them at home on their smartphones or laptops. The yoga-mindfulness intervention spanned 8-weeks, with one session per week lasting approximately 1 h (see *School-based Yoga-Mindfulness Intervention* below). The intervention was conducted by trained professionals (F.I. who holds a Yoga Bimbi Instructor certification recognized by the National Educational Sport Center [CSEN] accredited by the Italian National Olympic Committee [CONI], and L.S. who is an experienced yoga-mindfulness teacher without formal certifications), who are psychologists that completed a specialized course in yoga and mindfulness for children. The intervention took place in the school gymnasium during physical education hours, under the supervision of the teacher.

#### 2.4.1 School-based yoga-mindfulness intervention

Since one of the aims of the present study was to provide a practical protocol to implement a yoga-mindfulness intervention in Italian schools, below we outline a detailed description of each phase of the project.

Given the relevance of the school’s compliance for the success of the study, the whole research project was first presented by the NeuroDev research team to both the principal and the teaching board. Once agreed to participate, the school invited all the interested families to provide written, informed consent.

The intervention was structured into 8 sessions and based on the Mindfulness Based Stress Reduction protocol (MBSR; e.g., Kabat-Zinn, 1990, 2003). It included activities inspired by Yoga exercises, developed to be engaging for children, and differentiated by age groups but maintaining the same fundamental framework.

Each session included the following phases:

1. Introductory game. The aim of this phase was to let children become familiar with their own emotions and moods by providing a relationship-centered form of active attention and memorization. Practically, children form a circle around the yoga teacher. In turn, each child has to say his/her name, to verbally express his/her feelings, and make a gesture or movement that represents him/herself. Additionally, each child has to remember and repeat the movement displayed by the previous child.
2. Warm-up. During this phase, imaginative games of increasing physical intensity are created to offer a time of physical “unloading” that prepares the child for the following relaxation and increased stillness of the asanas (Yoga postures). Children take their seats on mattresses, which will now be the delimiters of the movement area (see Figure 3), allowing children to learn the management of their own space and the maintenance of limited mobility, which includes respecting others’ spaces.
3. Asanas. The first session included the reading of a tale about the origins of yoga in order to get the children more engaged. In the following sessions, asanas of increasing complexity were demonstrated, and children had to reproduce specific yoga positions cued by figurative cards shown by the yoga teacher. Then, dynamic games were also proposed, allowing children to consolidate the learnt card-position association.
4. Mindfulness. In this phase children were taught to use breathing and meditations techniques to achieve a state of mindfulness, meant as the capacity to remain in the “here and now”.
5. Relaxation. The final relaxation phase consists of listening to relaxing music while focusing on various parts of the body through a brief visualization exercise. The visualization is completed by adopting and maintaining the supine lying position for as long as possible.

Music, noises, and the use of instruments (i.e., Tibetan bell, steel tongue drum, etc.,) are provided throughout all the phases to increase the immersiveness of the experience.

### 2.5 Statistical analysis

The analyses were conducted using R software (R Core Team, 2021; version 4.1.3). Generalized Linear Mixed-effects models (GLMM) were fitted (R package: ‘lme4’, Bates, 2010), using family distributions that were selected based on the specific distribution characteristics of the data. GLMM allow the inclusion of both fixed and random effects, capturing individual-level variation and providing more precise estimates (Nakagawa and Schielzeth, 2013). Additionally, these models account for correlation structures within the data, increasing the model’s robustness and reducing the risk of spurious findings. Continuous numeric predictors were standardized before fitting GLMM to improve interpretability, ease convergence, mitigate multicollinearity, allow for easier effect comparisons, enhance model resilience against outliers, facilitate regularization, and ensure reproducibility. Models assumptions (i.e., homogeneity of variance, influential observations, collinearity, normality of residuals, normality of random effects) were assessed through visual exploration and posterior predictive checks (R package: ‘performance’, Lüdecke et al., 2021). The models were tested against the null model using the Akaike Information Criterion (AIC; R package: ‘stats’; R Core Team, 2021) to ensure that the predictors significantly improve model fit to the data (Cavanaugh and Neath, 2019) (see Supplementary materials S2). See Supplementary materials for model selection results.

The effects of GLMM were assessed using X^2^-test or *F*-test and *p*-values, calculated via Satterthwaite’s degrees of freedom method (*α* = 0.05, R package: ‘lmerTest’; Kuznetsova et al., 2017). *Post-hoc* pairwise comparisons between the levels of fixed factors were conducted using estimated marginal means (EMMs) contrasts, Bonferroni adjusted for multiple comparisons (R package: ‘emmeans’; Lenth, 2020). For each model, we reported the estimates with standard error (SE) and the associated statistics (*z*-test or *t*-test).

#### 2.5.1 The Dynamic Temporal Prediction task

Based on previous results (Del Popolo Cristaldi et al., 2023; Los et al., 2017; Mento and Granziol, 2020), who reported that CC adaptation was greater in short compared to long SOAs, we limited the analyses to short SOAs (400 ms) trials (12 trials in “slow blocks”, 24 trials in “fast blocks”). Indeed, these are the trials where we expect the hypothesized effect (i.e., RT adaptation to the global predictive context). The study had a 4 (block: slow-1, fast-1, slow-2, fast-2) x 2 (time: pre-yoga, post-yoga) within-subject design. Reaction times (RTs) were adjusted for the speed-accuracy trade-off by means of Inverse Efficiency Score (IES; Vandierendonck, 2017). The Inverse Efficiency Score is a well-known and consolidated index calculated by the following formula: RTs/(1–proportion of errors). The Inverse Efficiency Score was analyzed after removing premature (i.e., <150 ms before target onset, 3% of trials) and incorrect (i.e., omission or commission errors, 6% of trials) responses, resulting in 95% of trials remaining for analysis. Age (in years) was scaled before entering the models as a covariate. To test our hypotheses (H1a and H2a) we fitted the following GLMM:


$$IES\sim 1+{\mathrm{block}}^{*}\;\mathrm{time}+\mathrm{age}\;\left(\mathrm{years}\right)+\left(1\mathrm{|subj}\right),\mathrm{Gamma family distribution}$$

Dependent variable = IESIndependent variables = block × time + age (years)Random effect = participant Family distribution = Gamma

To assess H1a we run post-hoc pairwise contrasts comparing IES between the first two (slow-1 vs. fast-1) and last two (slow-2 vs. fast-2) blocks of the pre-yoga session. No IES difference or smaller IES in the “fast” compared to the “slow blocks” would suggest CC adaptation. To assess H1b, we will run the same contrasts on the post-yoga session. Moreover, we will run additional contrasts to directly compare the degree of early (slow-1 vs. fast-1) and late (slow-2 vs. fast-2) adaptation between the two sessions.

Analyses on accuracy and RTs are reported on Supplementary materials S3.

#### 2.5.2 Flanker task

In the analysis we did not consider the effect of the warning cue, as both the orienting and alerting effects were not of our interest for the purpose of the present study (see Table S8 for cue distributions and Table S9 for raw accuracy, RTs and IES across conditions). Therefore, the study had a 2 (block: non predictive, predictive) x 2 (trial: congruent, incongruent) x 2 (time: pre-yoga, post-yoga) within-subject design. To avoid multicollinearity issues, we created a single variable for block and trial (condition: congruent-NP, incongruent-NP, congruent-P, congruent-NP). Using the same procedure as in the DTP task, the IES was calculated to account for the speed-accuracy trade-off. Afterwards, premature responses (i.e., <150 ms before target onset, 2% of trials) and incorrect responses (i.e., omission or commission errors, 23% of trials) were removed, resulting in 81% of trials remaining for analysis. In order to disentangle CC adaptation from potential fatigue effects, we entered the trial number as a covariate. Age (in years) was scaled before entering the models. To test our hypotheses (H1b and H2b) we fitted the following GLMM:


$$\begin{array}{l} IES\sim 1+{\mathrm{condition}}^{*}\;\mathrm{time}+\mathrm{age}\;\left(\mathrm{years}\right)+\mathrm{trial number}\\ \;+\left(1\mathrm{|subj}\right),\mathrm{Gamma family distribution} \end{array}$$

Dependent variable = IESIndependent variables = condition × time + trial number + age (years)Random effect = participantFamily distribution = Gamma

To assess H2a we run *post-hoc* pairwise contrasts to compare the congruency effect (incongruent—congruent) in the non-predictive compared to the predictive block of the pre-yoga session. Smaller effects in the first block would suggest CC adaptation across the task. To assess H2b, we will run the same contrasts on the post-yoga session. Moreover, we will run additional contrasts to directly compare the degree of adaptation between the two sessions.

Analyses on accuracy and RTs are reported on Supplementary materials S3.

#### 2.5.3 Questionnaires

The study has a 2 (scales: CPRS_T, PSI_T) x 2 (time: pre-yoga, post-yoga) within-subject design. To test our hypothesis (H3) we fitted the following GLMM:


$$\mathrm{scores}\sim 1+{\mathrm{scales}}^{*}\;\mathrm{time}+\left(1\mathrm{|subj}\right),\mathrm{Poisson family distribution}$$

Dependent variable = scoresIndependent variables = scales × timeRandom effect = participantFamily distribution = Poisson

## 3 Results

### 3.1 The Dynamic Temporal Prediction task: inverse efficiency score

We found significant main effects of *block* [*X^2^*(3) = 307.81, *p* < 0.001], *time* [*X^2^*(1) = 149.12, *p* < 0.001], and a significant interaction *block* x *time* [*X^2^*(3) = 10.83, *p* = 0.001], but no significant main effect of *age (years)* [*X^2^*(1) = 1.31, *p* = 0.253].

First, we found an overall performance improvement as revealed by a decrease in IES in the post-yoga compared to the pre-yoga session, in all the blocks except for the third block (see Table 1 for post-hoc contrasts). Regarding specifically adaptation, as expected (H1a), only late adaptation occurred during the pre-yoga session as we found IES increase between the first two blocks [slow-1 vs. fast-1: –0.03, SE = 0.01, *z*_(Inf)_ = −3.6, *p* = 0.002] and no difference between the last two blocks [slow-2 vs. fast-2: –0.01, SE = 0.01, *z*_(Inf)_ = −1.44, *p* = 0.894]. In line with our hypothesis (H2a), in the post-yoga session we observed an optimization of both early and late CC adaptation. This was reflected by a stabilization of IES between the first two blocks [slow-1 vs. fast-1: –0.02, SE = 0.01, *z*_(Inf)_ = −2.56, *p* = 0.064] and a reduction of IES between the last two blocks [slow-2 vs. fast-2: 0.03, SE = 0.01, *z*_(Inf)_ = 3.10, *p* = 0.012]. When directly comparing the two sessions, we found a significant increase in the degree of late adaptation [pre-yoga(slow-2 vs. fast-2) vs. post-yoga(slow-2 vs. fast-2): 0.04, SE = 0.01, *z*_(Inf)_ = 3.2, *p* = 0.001] but no difference in early adaptation [pre-yoga (slow-1 vs. fast-1) vs. post-yoga (slow-1 vs. fast-1): 0.01, SE = 0.01, *z*_(Inf)_ = 0.7, *p* = 0.459] in the post-compared to the pre-yoga session.

**Table 1:** *Post-hoc* contrasts of the block * time interaction.

	Contrast	Estimate	SE	df	*z*	*p*
Slow-1	Pre-yoga vs. post-yoga	0.06	0.01	Inf	5.7	<0.001
Fast-1	Pre-yoga vs. post-yoga	0.07	0.01	Inf	9.1	<0.001
Slow-2	Pre-yoga vs. post-yoga	0.04	0.01	Inf	3.7	0.0002
Fast-2	Pre-yoga vs. post-yoga	0.08	0.01	Inf	10.3	<0.001
Pre-yoga	Slow-1 vs. Fast-1	-0.03	0.01	Inf	−3.6	0.002
Pre-yoga	Slow-2 vs. Fast-2	−0.01	0.01	Inf	−1.4	0.894
Post-yoga	Slow-1 vs. Fast-1	−0.02	0.01	Inf	−2.6	0.064
Post-yoga	Slow-2 vs. Fast-2	0.03	0.01	Inf	3.1	0.012

The table presents *post-hoc* contrasts of the block × time interaction, comparing the pre-yoga and post-yoga conditions across multiple contrasts. For each contrast, the table includes the contrast estimate, standard error (SE), degrees of freedom (df), *z*-statistic, and *p*-value. These contrasts help to highlight the impact of the yoga intervention on adaptive cognitive control, and the statistical significance is indicated for each contrast (with *p*-values <0.05 for most of the comparisons).

**Figure 4:**
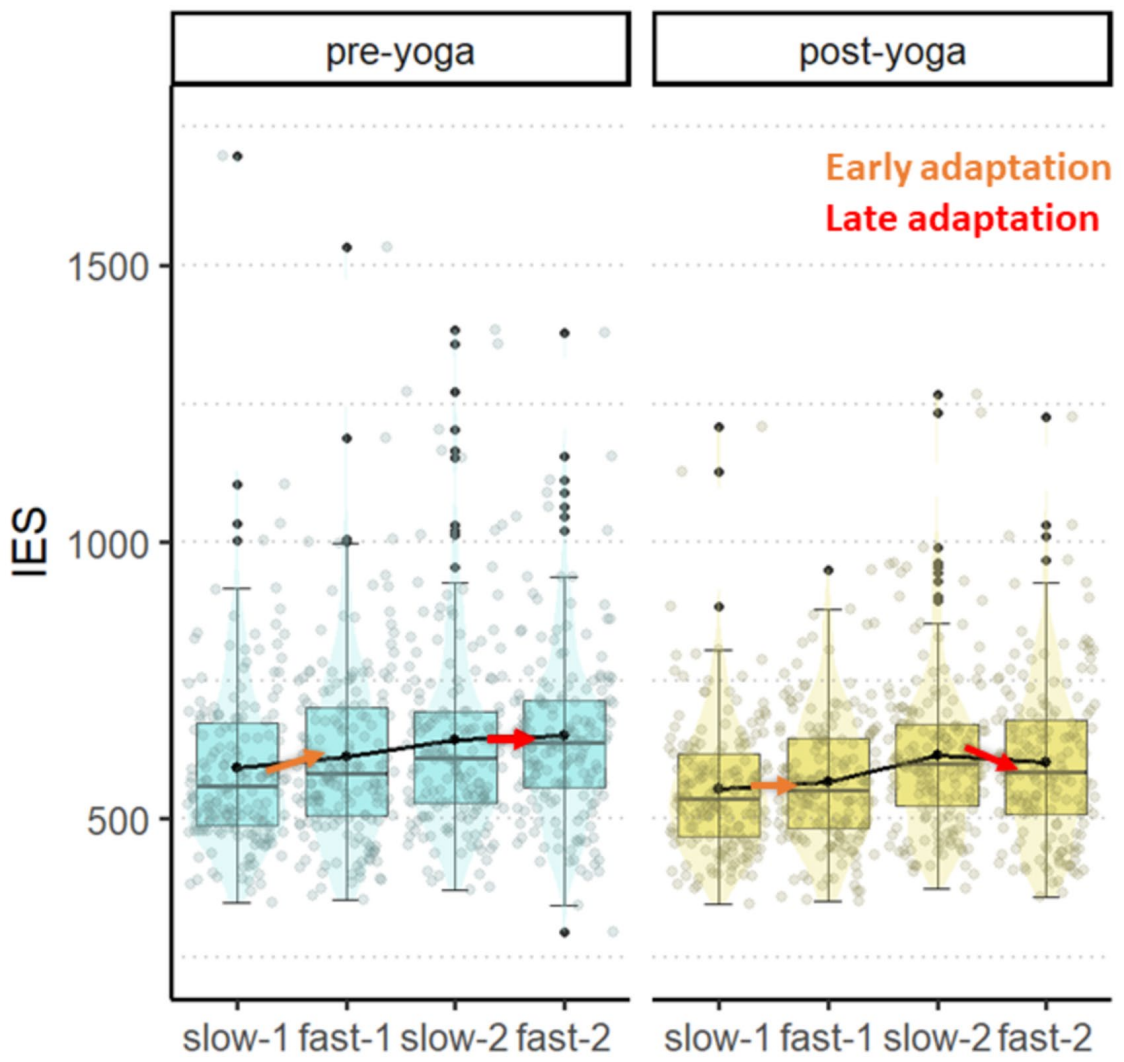
Inverse Efficiency Score (IES) in the DTP task. The plot displays IES on the y-axis along the four blocks in the x-axis (slow-1, fast-1, slow-2, fast-2), in the pre-yoga (left sub-panel) and post-yoga (right sub-panel) sessions. Arrows indicate the degree of adaptation in the first (slow-1 → fast-1; early adaptation) and second (slow-2 → fast-2; later adaptation) part of the task. Each box spans the interquartile range, with its edges indicating the first quartile and third quartile. The median is represented by a line inside the box. Additionally, individual observations are depicted as jittered points.

See Table 2 for raw accuracy, RTs and IES across conditions. Figure 4 for visual representation of the results.

**Table 2:** Raw accuracy, RTs and IES across conditions.

		Accuracy (mean ± sd)	RT (mean ± sd)	IES (mean ± sd)
Pre-yoga	Slow-1	0.96 ± 0.2	563 ± 202	586 ± 225
	Fast-1	0.94 ± 0.2	564 ± 217	605 ± 251
	Slow-2	0.94 ± 0.2	593 ± 220	633 ± 258
	Fast-2	0.93 ± 0.3	593 ± 228	641 ± 267
Post-yoga	Slow-1	0.98 ± 0.1	539 ± 171	551 ± 187
	Fast-1	0.95 ± 0.2	538 ± 193	565 ± 210
	Slow-2	0.96 ± 0.2	580 ± 202	608 ± 230
	Fast-2	0.94 ± 0.2	560 ± 214	595 ± 243

For each measure we reported the mean and standard deviation.

### 3.2 The Flanker task: inverse efficiency score

We found significant main effect of *condition* [*X*^2^(3) = 2439.6, *p* < 0.001], *time* [*X^2^*(1) = 566.1, *p* < 0.001], *age (years)* [*X^2^*(1) = 18.9, *p* < 0.001], a significant interaction *condition* x *time* [*X^2^*(3) = 105.6, *p* < 0.001] but no effect of *trial number* [*X*^2^(1) = 2.2, *p* = 0.138].

First, we found an overall performance improvement in the post-yoga compared to the pre-yoga session, as revealed by a reduction of IES in all the blocks (see Table 3 for post-hoc contrasts). Expected congruency effects were present in both the pre-yoga [non-predictive: 0.23, SE = 0.01, *z*(Inf) = 22.9, *p* < 0.001; predictive: 0.37, SE = 0.01, *z*_(Inf)_ = 26.8, *p* < 0.001] and post-yoga [non-predictive: 0.16, SE = 0.01, *z*_(Inf)_ = 16.7, *p* < 0.001; predictive: 0.34, SE = 0.01, *z*_(Inf)_ = 24.8, *p* < 0.001] sessions. Interestingly, the congruency effect decreased from the pre-yoga to the post-yoga session in the non-predictive block [pre-yoga vs. post-yoga: 0.06, SE = 0.01, *z*_(Inf)_ = 4.7, *p* < 0.001], suggesting better cognitive interference management.

**Table 3:** *Post-hoc* contrasts of the condition * time interaction.

	Contrast	Estimate	SE	df	*z*	*p*
Congruent B1	Pre-yoga vs. post-yoga	0.18	0.01	inf	16.6	<0.001
Congruent B2	Pre-yoga vs. post-yoga	0.11	0.01	inf	10.9	<0.001
Incongruent B1	Pre-yoga vs. post-yoga	0.24	0.01	inf	22.1	<0.001
Incongruent B2	Pre-yoga vs. post-yoga	0.14	0.02	inf	7.9	<0.001
Pre-yoga	Congruent B1 vs. congruent B2	−0.17	0.01	inf	−13.4	<0.001
Pre-yoga	Congruent B1 vs. incongruent B1	−0.23	0.01	inf	−22.9	<0.001
Pre-yoga	Congruent B2 vs. incongruent B2	−0.37	0.01	inf	−26.8	<0.001
Pre-yoga	Incongruent B1 vs. incongruent B2	−0.31	0.02	inf	−18.7	<0.001
Post-yoga	Congruent B1 vs. congruent B2	−0.24	0.01	inf	−18.9	<0.001
Post-yoga	Congruent B1 vs. incongruent B1	−0.16	0.01	inf	−16.7	<0.001
Post-yoga	Congruent B2 vs. incongruent B2	−0.34	0.01	inf	−24.8	<0.001
Post-yoga	Incongruent B1 vs. incongruent B2	−0.42	0.02	inf	−25.4	<0.001

The Table presents post-hoc contrasts of the block × time interaction, comparing the pre-yoga and post-yoga conditions across multiple contrasts. For each contrast, the table includes the contrast estimate, standard error (SE), degrees of freedom (df), *z*-statistic, and *p*-value. These contrasts help to highlight the impact of the yoga intervention on adaptive cognitive control, and the statistical significance is indicated for each contrast (with *p*-values <0.05 for most of the comparisons).

**Figure 5:**
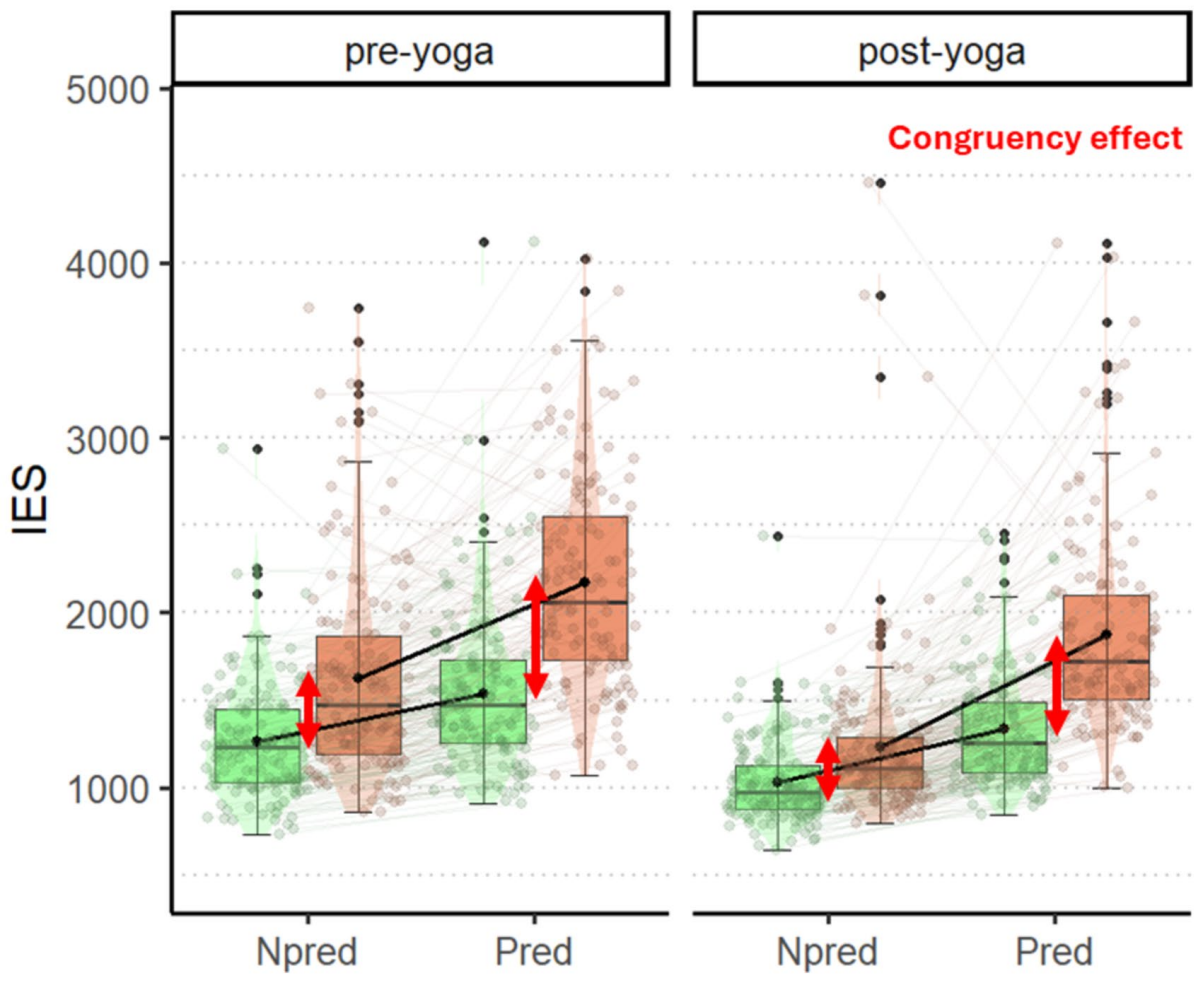
Inverse efficiency score (IES) in the Flanker task. The plot displays IES on the *y*-axis, along the two blocks in the *x*-axis (Npred, Pred), for both congruent (green) and incongruent (orange) trials in the pre-yoga (left sub-panel) and post-yoga (right sub-panel) sessions. Red arrows indicate the congruency effect in the Npred blocks in pre-and post-yoga sessions (smaller in the post-yoga session). Each box spans the interquartile range, with its edges indicating the first quartile and third quartile. The median is represented by a line inside the box. Additionally, individual observations are depicted as jittered points.

Regarding adaptation specifically, in line with our hypothesis (H2a), it occurred in both sessions. Indeed, the management of cognitive interference (i.e., congruency effect) was modulated in both pre-yoga [non-predictive vs. predictive: –0.14, SE = 0.02, *z*_(Inf)_ = −8.3, *p* < 0.001] and post-yoga [non-predictive vs. predictive: –0.18, SE = 0.02, *z*_(Inf)_ = −10.5, *p* < 0.001], being greater in the non-predictive compared to the predictive block. Specifically, in both sessions this modulation is the result of greater IES increase in incongruent than congruent trials across the blocks [pre-yoga congruent (predictive–non-predictive) vs. incongruent (predictive–non-predictive): –0.14, SE = 0.02, *z*_(Inf)_ = −8.3, *p* < 0.001; post-yoga congruent (predictive–non-predictive) vs. incongruent(predictive–non-predictive): –0.18, SE = 0.02, *z*_(Inf)_ = −10.5, *p* < 0.001]. However, differently from expected (H2b), the degree of this modulation did not differ between sessions [pre-yoga vs. post-yoga: 0.03, SE = 0.02, *z*_(Inf)_ = 1.5, *p* = 0.143]. See Table 4 for raw accuracy, RTs and IES across conditions and Figure 5 for visual representation of the results.

**Table 4:** Raw accuracy, RTs and IES across conditions.

		Accuracy (mean ± sd)	RT (mean ± sd)	IES (mean ± sd)
Pre-yoga	Congruent B1	0.93 ± 0.3	1,142 ± 377	1,249 ± 485
	Incongruent B1	0.86 ± 0.4	1,294 ± 420	1,573 ± 857
	Congruent B2	0.74 ± 0.4	1,092 ± 382	1,512 ± 629
	Incongruent B2	0.59 ± 0.5	1,251 ± 421	2,174 ± 980
Post-yoga	Congruent B1	0.95 ± 0.2	969 ± 304	1,021 ± 357
	Incongruent B1	0.92 ± 0.3	1,083 ± 339	1,206 ± 582
	Congruent B2	0.75 ± 0.4	970 ± 325	1,323 ± 552
	Incongruent B2	0.60 ± 0.5	1,091 ± 363	1844 ± 778

For each measure we reported the mean and standard deviation.

### 3.3 Questionnaires

**Figure 6:**
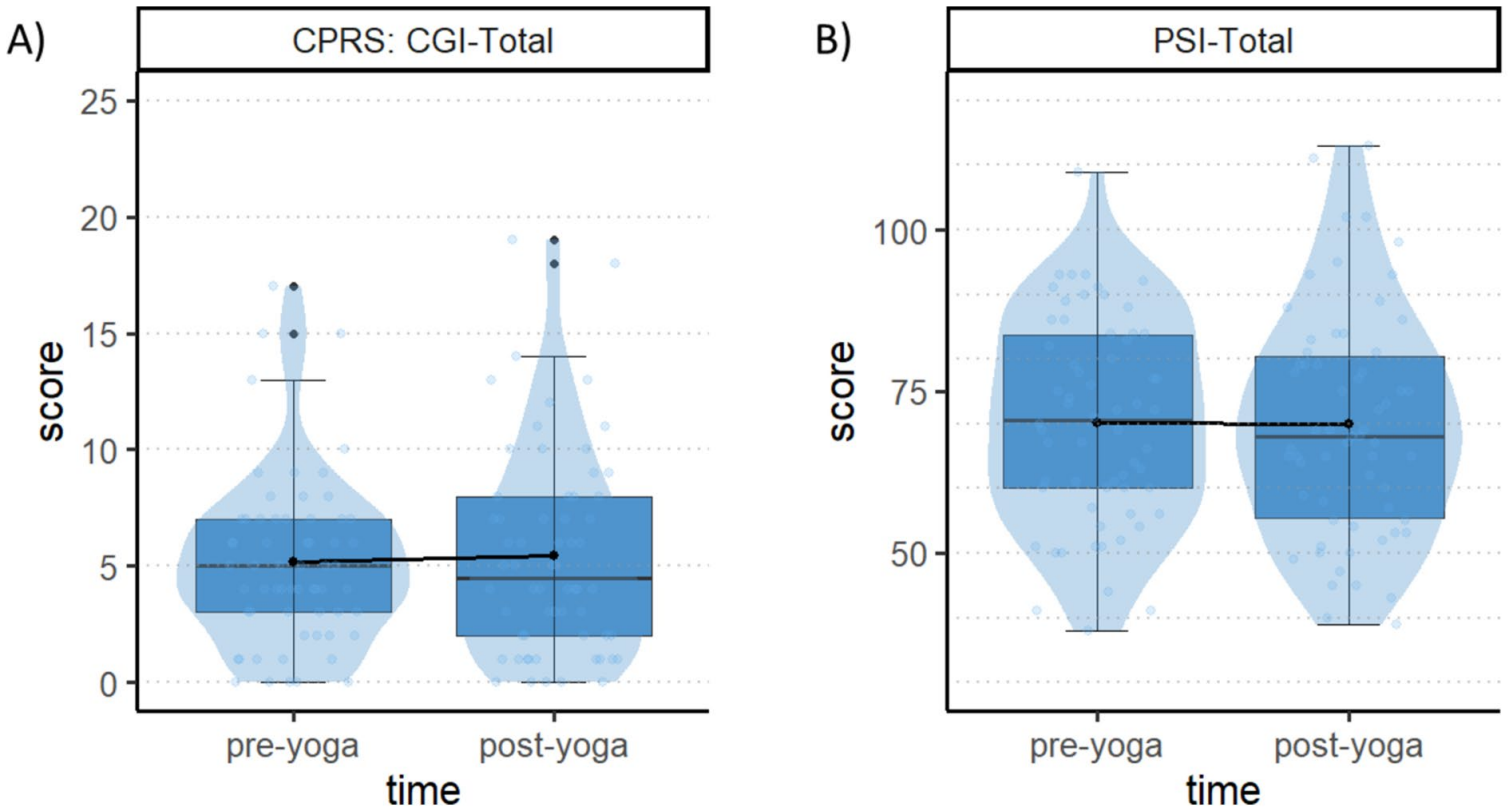
CPRS-R and PSI total scores. **(A)** The plot displays the CPRS-R CGI total scale scores on the *y*-axis, and the two pre-yoga and post-yoga sessions on the *x*-axis. **(B)** The plot displays the PSI total scale scores on the *y*-axis, and the two pre-yoga and post-yoga sessions on the *x*-axis. In both panels, each box spans the interquartile range, with its edges indicating the first quartile and third quartile. The median is represented by a line inside the box. Additionally, individual observations are depicted as jittered points.

Results revealed a main effect of *questionnaires’ scales* [*X^2^*(1) = 4086.43, *p* < 0.001], but no effect of *time* [*X^2^*(1) = 0.01, *p* = 0.923] nor of the interaction between questionnaires’ *scales and time* [*X^2^*(1) =0.54, *p* = 0.464]. Therefore, we did not find any difference between pre-yoga and post-yoga questionnaires’ scores. See Figure 6 for visual representation of the results.

## 4 Discussion

In the present study we investigated adaptive cognitive control (CC) in a group of preschool and school-aged children (4–7 years old). Specifically, we used the Dynamic Temporal Prediction task (DTP; Del Popolo Cristaldi et al., 2023; Mento and Granziol, 2020) and a modified version of the Flanker task (Gonthier et al., 2021; Gonthier and Blaye, 2021) to examine to what extent different predictive contexts contribute to the implicit adaptation of motor preparation/inhibition and cognitive interference, respectively. As a secondary goal, we assessed the feasibility of conducting a school-based yoga-mindfulness intervention in the Italian context and, for the first time, we evaluated its effectiveness in enhancing adaptive CC.

### 4.1 Adaptive cognitive control in young children

Previous findings showed that adaptive CC is already in place from preschool age (Chevalier et al., 2020; Gonthier et al., 2021), but the ability to regulate reactive and proactive control mechanisms shows a protracted development until adulthood (Del Popolo Cristaldi et al., 2023). Therefore, it is possible that the two different CC mechanisms exhibit dissociable developmental trajectories. Here, we propose two tasks that tap into different aspects of adaptive CC: the DTP and the modified Flanker task.

The Dynamic Temporal Prediction task was previously employed to illustrate developmental trajectories of motor preparation and inhibition adaptation to block-wise temporal predictive changes. As the main result, in the pre-intervention assessment we confirmed that young children (6–7 years old) do exhibit CC adaptation (Del Popolo Cristaldi et al., 2023), and we extended this finding to preschool-aged children (4–5 years old). Specifically, we found that their performance remained relatively stable between the first two (slow-1, fast-1) and last two (slow-2, fast2) blocks, suggesting that the task’s predictive context counterbalanced the otherwise expected fatigue effect along the task. Alongside, the modified Flanker task allowed us to investigate the adaptation of interference control to different predictive contexts. Our results not only confirmed previous reports of adaptive CC in children (Gonthier et al., 2021), but also extended these results to 4-years old children. We found that children modulated the congruency effect (i.e., incongruent vs. congruent speed-accuracy trade-off) along the task, demonstrating a reduction in the non-predictive (50% congruent trials) compared to the predictive (75% congruent trials) block. This suggests that even children from a very young age can engage CC proactively when implicit contextual contingencies make the use of reactive control more difficult or disadvantageous (as in the non-predictive block). At the same time, they flexibly shift to a more reactive CC modality when the context requires less active monitoring (as in the predictive block). In fact, it is more convenient to recruit cognitive resources when needed, rather than maintaining them active when not often required (given that incongruent trials are fewer than congruent trials in the predictive block). However, accuracy significantly dropped in the predictive block, suggesting that the on-the-fly recruitment of cognitive resources is still not optimized at this age. This could depend on a less efficient regulation of excitatory and inhibitory motor instances in the younger children (Bedard et al., 2002; Lewis et al., 2017). We suggest that with development the efficiency of adaptive CC increases in terms of greater ability to maintain high levels of accuracy while adjusting reaction times. Overall, these results confirm that mechanisms supporting adaptive CC are already in place at least from preschool age.

### 4.2 Yoga-mindfulness promotes different shades of adaptive cognitive control

As a secondary goal, we assessed the feasibility of a school-based yoga-mindfulness intervention in Italy specifically aimed at targeting its impact on adaptive CC. Indeed, the literature suggests that these practices are effective not only in enhancing general mental health (e.g., reduced stress; Chiesa and Serretti, 2009) but also in improving CC (Incagli et al., 2020). However, little is known about their potential effects on a more dimensional aspect of CC, like its ability to adapt to changing environmental contingencies. Firstly, our study demonstrated that implementing this kind of intervention (i.e., an 8-week yoga-mindfulness intervention for children held by trained professionals with an initial and final evaluation session) is feasible as an integrative activity of standard Italian schools’ curricula, even during challenging periods such as the covid-19 pandemic. Secondly, our results suggest potential positive effects of yoga-mindfulness on adaptive CC.

In terms of the DTP task, all children exhibited later adaptation during the final evaluation session. Specifically, they responded faster while maintaining accuracy from the third (slow-2) to the fourth (fast-2) block, indicating that they inferred the global predictive structure. This implies that this practice might facilitate the early establishment of an adaptation that is typically shown to become spontaneously efficient from adolescence (Del Popolo Cristaldi et al., 2023). Regarding the Flanker task, we found that cognitive interference in terms of speed-accuracy trade-off was reduced in the non-predictive block, but not in the predictive one, during the final evaluation. Therefore, we suggest that yoga-mindfulness interventions might enhance children’s cognitive conflict resolution only when control is proactively engaged in a high-conflict predictive context. Taken together, the post-intervention behavioral changes we observed seem to suggest an increased ability to efficiently manage CC in terms of adaptation regarding both motor response preparation/inhibition (DTP task) and interference control (Flanker task). More specifically, a possible common mechanism guiding the behavioral optimization could be related to the putative role of yoga-mindfulness interventions on improving self-regulation and attentional control through an increased mind–body awareness (Crescentini et al., 2016; Malinowski, 2013). This latter may promote the adaptive use of CC through a more efficient engagement of proactive modalities as a function of contextual demands. Moreover, our questionnaire results suggest that the intervention prevented potential negative effects of the covid-19 pandemic. Indeed, many studies showed that pandemic-related consequences (e.g., health issues, social restrictions) negatively affected children’s behavior and emotion regulation as assessed using parental questionnaires (Bonvino et al., 2023). Thus, the absence of significant differences in the questionnaires’ scores (CPRS; Conners, 2001; Nobile et al., 2007; PSI; Abidin, 1995; Guarino et al., 2008), before and after intervention, could suggest positive compensatory effects on children’s well-being.

### 4.3 Limitations and future directions

The main limitation of the study consists in the absence of a control group, since all children included in this research participated in the yoga-mindfulness intervention.

Given the prevailing pandemic circumstances, the researchers opted not to preclude any individuals from receiving the intervention, recognizing its potential benefits for their well-being. Consequently, all participants were administered the intervention, and resource limitations prevented the recruitment of a control group post-research completion. Therefore, we cannot draw firm conclusions about the yoga-mindfulness intervention effects on adaptive CC. Nevertheless, results suggest that differences in the post-compared to the pre-intervention do not regard an overall performance improvement associated with a practice effect. Instead, we found improvements related to the task-specific internal manipulation that cannot easily be explained by a mere effect of practice. In fact, the time elapsed between the two sessions should have prevented retrieval of the tasks’ statistical associations on which these improvements depend on. We encourage future research to overcome these limitations by replicating our results using an active control group performing alternative activities than yoga-mindfulness.

Another potential limitation is the block order of the Flanker task (non-predictive → predictive). Indeed, the expected CC adaptation effects (performance decrease in the predictive block) could easily be confounded with fatigue effects. Although we controlled for this potential issue, future studies should replicate our findings with the blocks presented in reverse order to further validate the results.

## Data Availability

The datasets presented in this study can be found in online repositories. The names of the repository/repositories and accession number(s) can be found a: https://osf.io/kv67j/?view_only=769dda38437048b3b1329783bc1499f6.
