# Supplementary material for: Adaptive cognitive control in 4 to 7-year-old children and potential effects of school-based yoga-mindfulness interventions: an exploratory study in Italy
Source: Front Psychol. 2025 Jan 24;16:1379241. doi: 10.3389/fpsyg.2025.1379241 (PMC11803634; doi:10.3389/fpsyg.2025.1379241)
Supplement: Supplementary file 2 [file Data_Sheet_2.PDF]

Supplementary material

S1. Sample

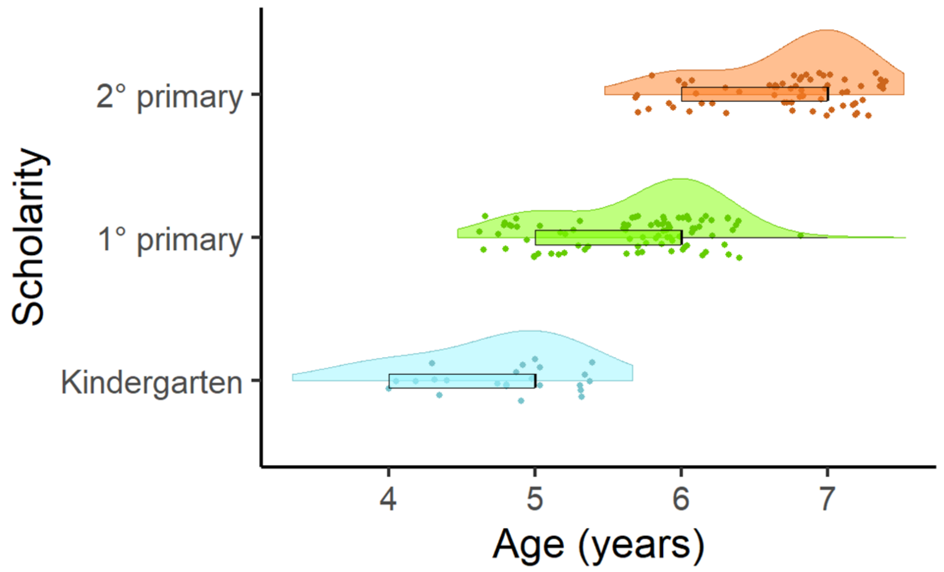

Figure S1. DTP final sample. Raincloud plot and boxplot of participants' age (years) distribution in Kindergarten, 1° primary school and 2° primary school.

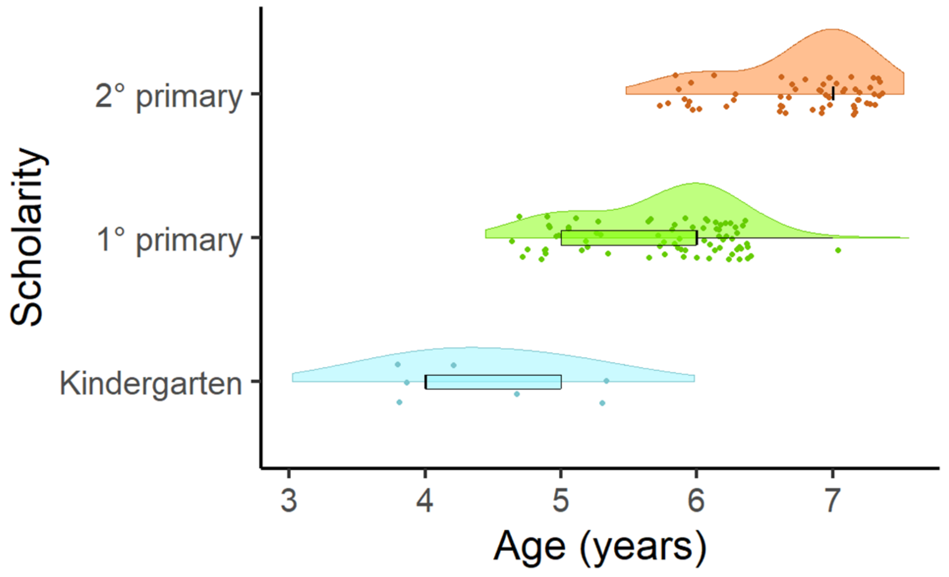

Figure S2. Flanker final sample. Raincloud plot and boxplot of participants' age (years) distribution in Kindergarten, 1° primary school and 2° primary school.

S2. Model selection

Below we present a comparative analysis of different models using the Akaike Information Criterion (AIC) as a measure of model fit. A lower AIC value indicates a better fit to the data. In our selection process, preference is given to models with lower AIC scores. If two or more models exhibit identical AIC values, the criterion for model selection prioritizes parsimony. The more parsimonious model, characterized by a simpler structure or fewer parameters, is favored. This approach ensures that model selection not only considers goodness of fit but also adheres to the principle of preferring simpler explanations when faced with equivalent explanatory power.

Dynamic Temporal Prediction (DTP) task

Variables description:

- age (years) → continuous variable, scaled
- block → factor variable, levels: slow-1, fast-1, slow-2, fast-2
- time → factor variable, levels: pre-yoga, post-yoga
- accuracy → numeric variable, 0 = incorrect, 1 = correct
- RT (reaction times) → numeric continuous variable
- IES (inverse efficiency score) → numeric continuous variable, calculated as RT/(1-proportion of errors)

Accuracy

The following Generalized Linear Mixed-effects Models (GLMMs) were run using the Binomial family distribution:

ACC_M_null: accuracy ~ 1 + (1|id)

ACC_M_1: accuracy ~ 1 + block * time + age (years) + (1|id)

**Model**	**df**	**AIC**
ACC_M_null	2	11239.89
**ACC_M_1**	**3**	**11123.40**

Table S1. Model selection results. For each model, the table reports degrees of freedom and the Akaike Information Criterion (AIC). A lower AIC indicates a model that better balances goodness of fit and complexity, minimizing overfitting while effectively capturing the underlying patterns in the data.

Reaction times (RTs)

The following Generalized Linear Mixed-effects Models (GLMMs) were run using the Gamma family distribution:

RT_M_null: RT ~ 1 + (1|id)

RT_M_1: RT ~ 1 + block * time + age (years) + (1|id)

**Model**	**df**	**AIC**
RT_M_null	3	345264.3
**RT_M_1**	**11**	**344937.2**

Table S2. Model selection results. For each model, the table reports degrees of freedom and the Akaike Information Criterion (AIC). A lower AIC indicates a model that better balances goodness of fit and complexity, minimizing overfitting while effectively capturing the underlying patterns in the data.

Inverse Efficiency Score (IES)

The following Linear Mixed-effects Models (LMMs) were run:

IES_M_null: log(IES) ~ 1 + (1|id)

IES_M_4: log(IES) ~ 1 + block * time + age (years) + (1|id)

**Model**	**df**	**AIC**
IES_M_null	3	349438.4
**IES_M_1**	**11**	**348886.1**

Table S3. Model selection results. For each model, the table reports degrees of freedom and the Akaike Information Criterion (AIC). A lower AIC indicates a model that better balances goodness of fit and complexity, minimizing overfitting while effectively capturing the underlying patterns in the data.

<u>Flanker task</u>

Variables description:

- age (years) → continuous variable, scaled
- condition→ factor variable, levels: congruentB1, congruentB2, incongruentB1, incongruentB2
- time → factor variable, levels: pre-yoga, post-yoga

- accuracy → numeric variable, 0 = incorrect, 1 = correct
- IES → numeric continuous variable, calculated as RT/(1-proportion of errors)
- trial number → continuous variable, scaled

Accuracy

The following Generalized Linear Mixed-effects Models (GLMMs) were run using the Binomial family distribution:

ACC_M_null: accuracy ~ 1 + (1|id)

ACC_M_1: accuracy ~ 1 + condition * time + age (years) + (1|id)

**Model**	**df**	**AIC**
ACC_M_null	2	17038.25
**ACC_M_1**	**11~~0~~**	**15403.79**

Table S4. Model selection results. For each model, the table reports degrees of freedom and the Akaike Information Criterion (AIC). A lower AIC indicates a model that better balances goodness of fit and complexity, minimizing overfitting while effectively capturing the underlying patterns in the data.

Reaction times (RTs)

The following Generalized Linear Mixed-effects Models (GLMMs) were run using the Gamma family distribution:

RT_M_null: RT ~ 1 + (1|id)

RT_M_1: RT ~ 1 + condition* time + age (years) + (1|id)

Model	df	AIC
RT_M_null	3	201900.2
**RT_M_1**	**12**	**200148.2**

Table S5. Model selection results. For each model, the table reports degrees of freedom and the Akaike Information Criterion (AIC). A lower AIC indicates a model that better balances goodness of fit and complexity, minimizing overfitting while effectively capturing the underlying patterns in the data.

Inverse Efficiency Score (IES)

The following Linear Mixed-effects Models (LMMs) were run:

IES_M_null: log(IES) ~ 1 + (1|id)

IES_M_1: log(IES) ~ 1 + condition * time + age (years) + (1|id)

Model	df	AIC
IES_M_null	3	214413.5
**IES_M_1**	**12**	**209944.6**

Table S6. Model selection results. For each model, the table reports degrees of freedom and the Akaike Information Criterion (AIC). A lower AIC indicates a model that better balances goodness of fit and complexity, minimizing overfitting while effectively capturing the underlying patterns in the data.

S3. Results

Dynamic Temporal Prediction (DTP) task

Accuracy

Results revealed a main effect of block ($X^2(3) = 78.66$, $p < .001$) and time ($X^2(1) = 15.86$, $p < .001$) but no effect of block x time ($X^2(3) = 3.32$, $p = .344$) or age (years) ($X^2(1) = 3.06$, $p = .080$; see Figure S3 for model predicted values). First, we found an overall performance improvement as revealed by an accuracy increase in the post-yoga compared to the pre-yoga in all the blocks except for the slow-2 block (see Table x for post-hoc results). Regarding block-by-block accuracy, we found an accuracy decrease from the first to the second block in both the pre-yoga (slow-1 vs. fast-1: 0.58, SE = 0.13, $z_{(Inf)} = 4.5$, $p < .001$) and post-yoga (slow-1 vs. fast-1: 0.86, SE = 0.17, $z_{(Inf)} = 5.1$, $p < .001$) session. Conversely, accuracy between the last two blocks remained stable in both the pre-yoga (slow-2 vs. fast-2: 0.22, SE = 0.11, $z_{(Inf)} = 2.0$, $p = .245$) and post-yoga (slow-2 vs. fast-2: 0.29, SE = 0.12, $z_{(Inf)} = 2.4$, $p = .106$) sessions.

	contrast	estimate	SE	df	z	p
Slow-1	pre-yoga vs post-yoga	-0.56	0.19	Inf	-2.88	0.004~~80~~
Fast-1	pre-yoga vs post-yoga	-0.28	0.10	Inf	-2.89	0.004~~32~~
Slow-2	pre-yoga vs post-yoga	-0.26	0.15	Inf	-1.76	0.078~~685~~
Fast-2	pre-yoga vs post-yoga	-0.19	0.09	Inf	-2.06	0.040~~69~~

pre-yoga	Slow-1 vs Fast-1	0.58	0.13	Inf	4.5	<.001
pre-yoga	Slow-2 vs Fast-2	0.22	0.11	Inf	2.0	.245
post-yoga	Slow-1 vs Fast-1	0.86	0.17	Inf	5.1	<.001
post-yoga	Slow-2 vs Fast-2	0.29	0.12	Inf	2.4	.106

Table S7. Post hoc contrasts of the block*time interaction of the ACC_M_1 model.

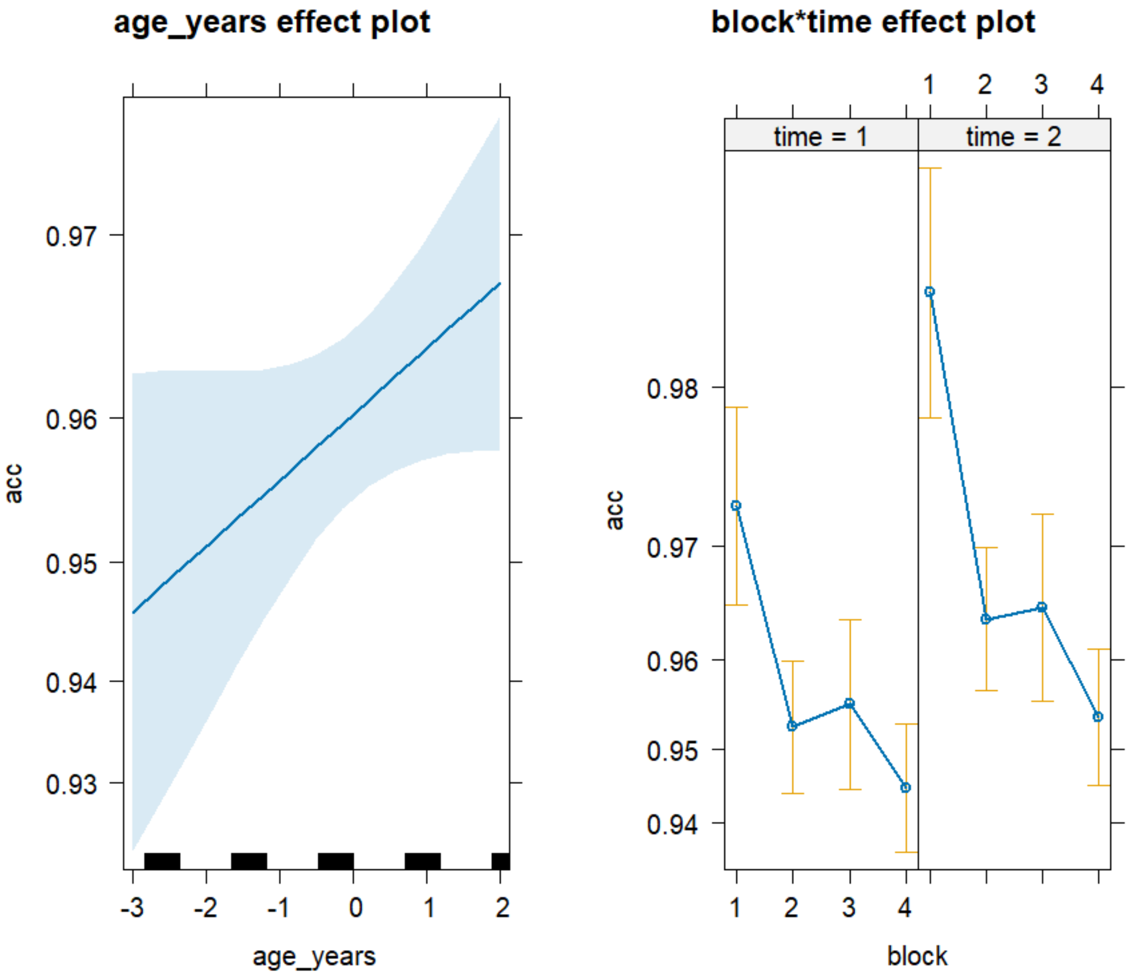

Figure S3. Results of the block * time interaction.

Reaction Times

Results revealed a main effect of block ($X^2(3) = 188.9$, $p < .001$), time ($X^2(1) = 56.5$, $p <.001$), block x time ($X^2(3) = 11.2$, $p =.011$) and age (years) ($X^2(1) = 2.7$, $p =.099$; see Figure S4 for model predicted values).

First, we found an overall performance improvement as revealed by an RTs decrease in the post-yoga compared to the pre-yoga in all the blocks (see Table S8 x for post-hoc results).

In terms of block-by-block RTs, in the pre-yoga session RTs remained stable from the first to the second block (slow-1 vs fast-1: -0.002, SE = 0.01, $z_{(Inf)} = -0.3$, $p =1.000$) and from the third to the fourth block (slow-2 vs fast-2: 0.002, SE = 0.01, $z_{(Inf)} = 0.3$, $p =1.000$). Instead, in the post-yoga session RTs remained stable between the first two blocks (slow-1 vs fast-1: 0.01, SE = 0.01, $z_{(Inf)} = 0.6$, $p =1.000$) but decreased between the last two blocks (slow-2 vs fast-2: 0.04, SE = 0.01, $z_{(Inf)} = 4.9$, $p <.001$).

	contrast	estimate	SE	df	z	p
Slow-1	pre-yoga vs post-yoga	0.03	0.01	Inf	3.2	.002
Fast-1	pre-yoga vs post-yoga	0.04	0.01	Inf	5.4	<.001
Slow-2	pre-yoga vs post-yoga	0.02	0.01	Inf	1.4	.165
Fast-2	pre-yoga vs post-yoga	0.05	0.01	Inf	7.1	<.001
pre-yoga	Slow-1 vs Fast-1	-0.002	0.01	Inf	-0.3	1.000

pre-yoga	Slow-2 vs Fast-2	0.002	0.01	Inf	0.3	1.000
post-yoga	Slow-1 vs Fast-1	0.01	0.01	Inf	0.6	1.000
post-yoga	Slow-2 vs Fast-2	0.04	0.01	Inf	4.9	<.001

Table S8. Post hoc contrasts of the block*time interaction of the RT_M_1 model.

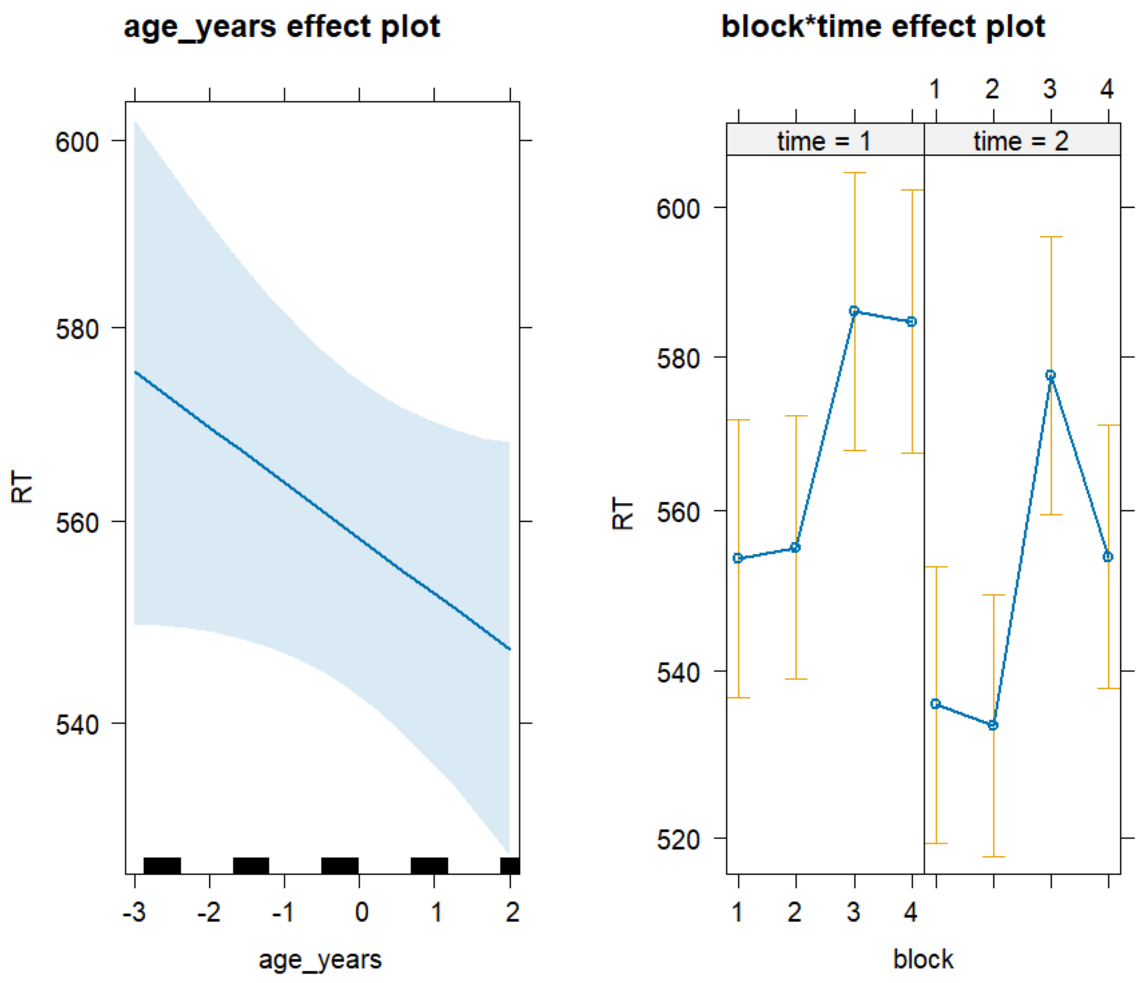

Figure S4. Results of the block * time interaction.

For a graphical representation of the data please see Figure S5.

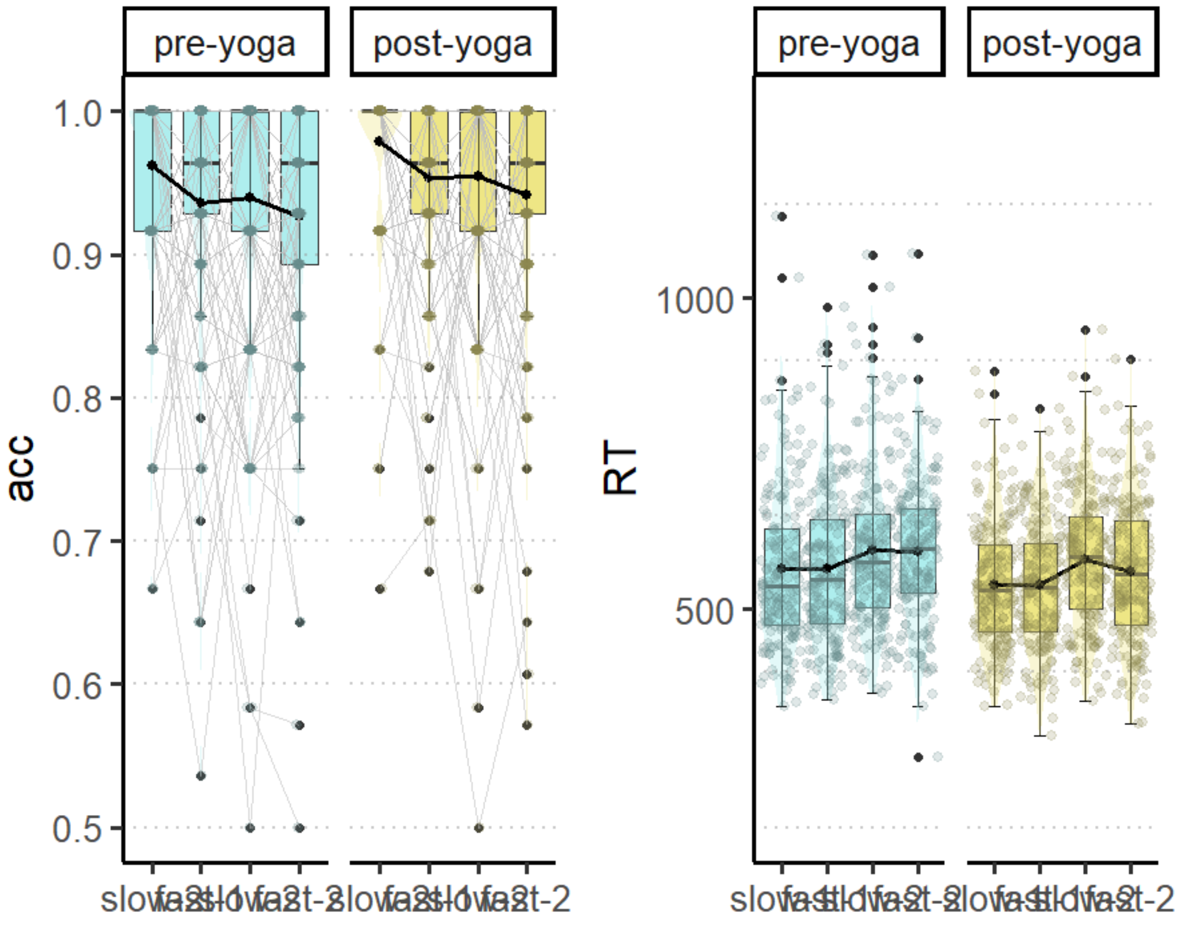

Figure S5. Accuracy and RTs in the DTP task. Panel A. The plot displays accuracy on the y-axis along the four blocks in the x-axis (slow-1, fast-1, slow-2, fast-2), in the pre-yoga (left sub-panel, light blue) and post-yoga (right sub-panel, light yellow) sessions. Panel B. The plot displays RT on the y-axis along the four blocks in the x-axis (slow-1, fast-1, slow-2, fast-2), in the pre-yoga (left sub-panel, light blue) and post-yoga (right sub-panel, light yellow) sessions. In both panels, each box spans the interquartile range, with its edges indicating the first quartile and third quartile. The median is represented by a line inside the box. Additionally, individual observations are depicted as jittered points.

IES

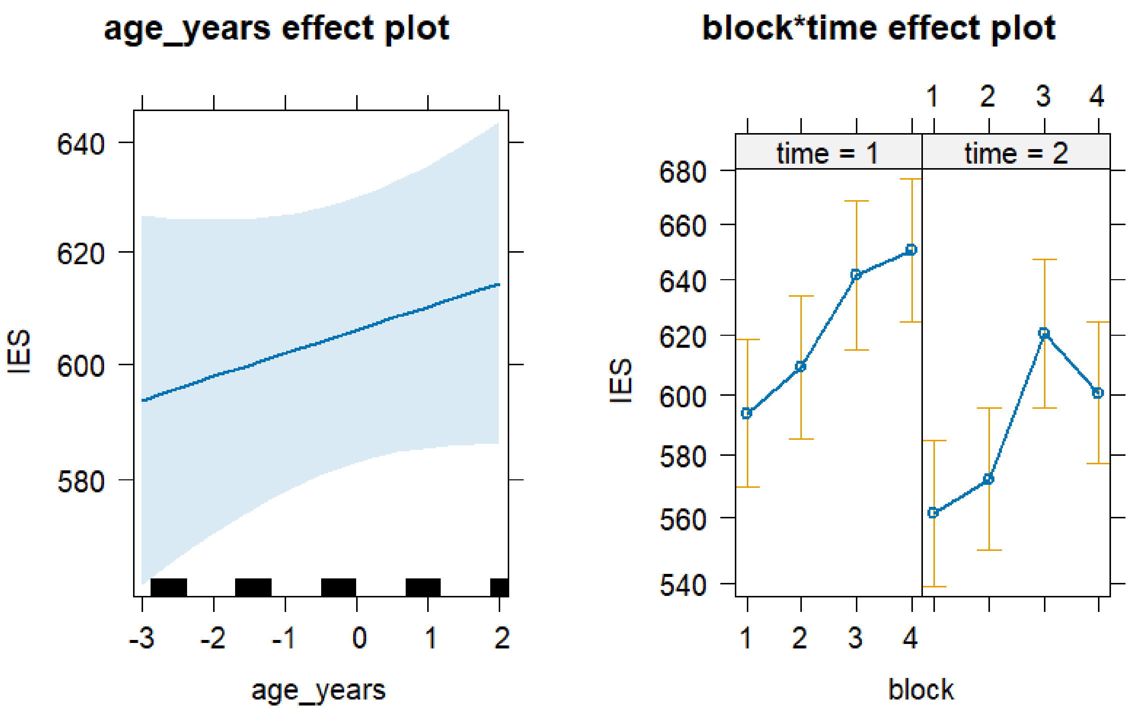

Figure S6. Results of the block * time interaction.

<u>Flanker task</u>

WarningType	TrialType	block	N° trial
up	congruent	nont predictive	4
		predictive	6
	incongruent	nont predictive	4
		predictive	2

down	congruent	non~~t~~ predictive	4
		predictive	6
	incongruent	non~~t~~ predictive	4
		predictive	2
center	congruent	non~~t~~ predictive	4
		predictive	6
	incongruent	non~~t~~ predictive	4
		predictive	2
no	congruent	non~~t~~ predictive	4
		predictive	6
	incongruent	non~~t~~ predictive	4
		predictive	2

Table S9. Distribution of warning cues across conditions.

**condition**	**time**	**warning**	**accuracy (mean ± sd)**	**RT (mean ± sd)**	**IES (mean ± sd)**
congruent B1	1	center~~no~~	0.93 ± 0.3	1165~~1171~~ ± 400~~410~~	1260 ± 484
congruent B1	1	down~~center~~	0.93 ± 0.3	1087~~1113~~ ± 351~~406~~	1196 ± 470
congruent B1	1	no~~up~~	0.93 ± 0.3	1153~~1168~~ ± 390~~422~~	1259 ± 504
congruent B1	1	up~~down~~	0.93 ± 0.3	1162~~1189~~ ± 361~~413~~	1280 ± 479
congruent B1	2	center~~no~~	0.97 ± 0.2	963~~1~~ ± 290~~1~~	1012 ± 344
congruent B1	2	down~~center~~	0.96 ± 0.2	956~~61~~ ± 324~~34~~	1011 ± 381
congruent B1	2	no~~up~~	0.95 ± 0.2	986~~8~~ ± 301~~12~~	1035 ± 344
congruent B1	2	up~~down~~	0.94 ± 0.2	970~~79~~ ± 300~~28~~	1024 ± 359
congruent B2	1	center~~no~~	0.77 ± 0.4	1081~~99~~ ± 375~~410~~	1487 ± 592

congruent B2	1	down~~center~~	0.78 ± 0.4	1077~~95~~ ± 378~~409~~	1492 ± 626
congruent B2	1	no~~up~~	0.63 ± 0.5	1103~~25~~ ± 381~~418~~	1527 ± 639
congruent B2	1	up~~down~~	0.78 ± 0.4	1107~~30~~ ± 393~~429~~	1544 ± 659
congruent B2	2	center~~no~~	0.79 ± 0.4	968~~999~~ ± 322~~404~~	1334 ± 588
congruent B2	2	down~~center~~	0.80 ± 0.4	943~~958~~ ± 327~~69~~	1275 ± 532
congruent B2	2	no~~up~~	0.64 ± 0.5	986~~1010~~ ± 330~~386~~	1341 ± 544
congruent B2	2	up~~down~~	0.79 ± 0.4	988~~1012~~ ± 322~~377~~	1348 ± 539
incongruent B1	1	center~~no~~	0.84~~5~~ ± 0.4	1328~~67~~ ± 434~~86~~	1592 ± 741
incongruent B1	1	down~~center~~	0.87~~8~~ ± 0.3	1258~~1306~~ ± 415~~93~~	1561 ± 974
incongruent B1	1	no~~up~~	0.84~~5~~ ± 0.4	130~~4~~8 ± 42~~8~~1	1605 ± 972

incongruent B1	1	up~~down~~	0.86 ± 0.3	1283~~1314~~ ± 406~~458~~	1534 ± 699
incongruent B1	2	center~~no~~	0.91 ± 0.3	1080~~95~~ ± 331~~69~~	1205 ± 613
incongruent B1	2	down~~center~~	0.92 ± 0.3	1076~~89~~ ± 352~~86~~	1196 ± 597
incongruent B1	2	no~~up~~	0.91~~2~~ ± 0.3	1081~~107~~ ± 330~~394~~	1215 ± 604
incongruent B1	2	up~~down~~	0.92~~3~~ ± 0.3	1094~~1102~~ ± 342~~366~~	1208 ± 513
incongruent B2	1	center~~no~~	0.50~~1~~ ± 0.5	1208~~63~~ ± 383~~470~~	2130 ± 933
incongruent B2	1	down~~center~~	0.49 ± 0.5	1240~~1240~~ ± 413	2079 ± 819
incongruent B2	1	no~~up~~	0.87~~8~~ ± 0.3	1279~~1320~~ ± 433~~490~~	2244 ± 1067
incongruent B2	1	up~~down~~	0.50 ± 0.5	1256~~1288~~ ± 445~~489~~	2192 ± 1011
incongruent B2	2	center~~no~~	0.49~~50~~ ± 0.5	1061~~1098~~ ± 326~~407~~	1829 ± 806

incongruent B2	2	down~~center~~	0.48 ± 0.5	1039~~48~~ ± 377~~406~~	1707 ± 672
incongruent B2	2	no~~up~~	0.92 ± 0.3	1146~~1159~~ ± 389~~416~~	1919 ± 755
incongruent B2	2	up~~down~~	0.51 ± 0.5	1073~~1086~~ ± 325~~351~~	1852 ± 870

Table S10. Raw RTs, accuracy and IES in each condition of the Flanker task. For each measure we report mean and standard deviation.

Accuracy

Results revealed a main effect of condition ($X^2(3) = 567.1$, $p < .001$), time ($X^2(1) = 13.4$, $p < .001$), age ($X^2(1) = 4.5$, $p = .034$), a significant interaction condition x time ($X^2(3) = 27.84$, $p < .001$) but no effects of trial number ($X^2(1) = 0.5$, $p =.462$; see Figure S3 for model predicted values).

First, we found an overall performance improvement in the post-yoga compared to the pre-yoga in the non~~t~~ predictive block; instead, no differences emerged in the predictive block (see Table S11 x for post-hoc results). Overall, as expected accuracy was greater for congruent compared to incongruent trials in both sessions and blocks. In terms of block-by-block performance, accuracy dropped from the non~~t~~ predictive to the predictive block in both the pre-yoga (congruent(non~~t~~ predictive - predictive: 1.6, SE = 0.1, $z_{(Inf)} = 13.3$, p<.001; incongruent(non~~t~~ predictive - predictive: 1.4, SE = 0.1, $z_{(Inf)} = 12.7$, p<.001) and post-yoga (congruent(non~~t~~ predictive - predictive: 1.9, SE = 0.1, $z_{(Inf)} = 14.5$, p<.001; incongruent(non~~t~~ predictive - predictive: 2.0, SE = 0.1, $z_{(Inf)} = 16.2$, p<.001) sessions.

	contrast	estimate	SE	df	z	p
congruent B1	pre-yoga vs post-yoga	-0.38	0.1	Inf	-2.81	0.005
congruent B2	pre-yoga vs post-yoga	-0.04	0.1	Inf	-0.74	0.463
incongruent B1	pre-yoga vs post-yoga	-0.58	0.1	Inf	-5.84	<.001
incongruent B2	pre-yoga vs post-yoga	-0.03	0.1	Inf	-0.29	0.771
pre-yoga	congruent B1 vs congruent B2	1.56	0.1	Inf	13.3	<.001
pre-yoga	congruent B1 vs incongruent B1	0.87	0.11	Inf	8.3	<.001
pre-yoga	congruent B2 vs incongruent B2	0.71	0.1	Inf	9.7	<.001
pre-yoga	incongruent B1 vs incongruent B2	1.41	0.1	Inf	12.7	<.001
post-yoga	congruent B1 vs congruent B2	1.90	0.1	Inf	14.5	<.001
post-yoga	congruent B1 vs incongruent B1	0.66	0.1	Inf	5.2	<.001
post-yoga	congruent B2 vs incongruent B2	0.73	0.1	Inf	9.8	<.001
post-yoga	incongruent B1 vs incongruent B2	2.00	0.1	Inf	16.2	<.001

Table S11. Post-hoc contrasts of the block * time interaction of the ACC_M_1 model

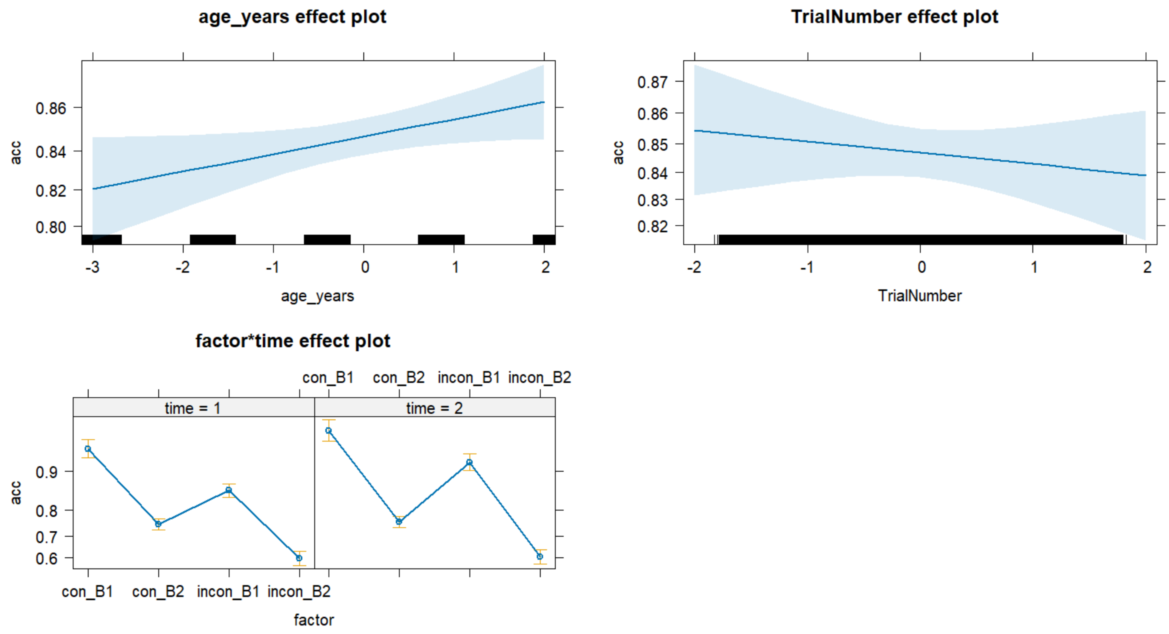

Figure S7. Results of the condition * time interaction.

Reaction Times

Results revealed a main effect of *condition* ($X^2(3) = 682.7$, $p < .001$), time ($X^2(1) = 442.2$, $p < .001$), *age* ($X^2(1) = 21.5$, $p < .001$), an interaction effect of *condition* x time ($X^2(3) = 34.4$, $p < .001$) but no effect of *trial number* ($X^2(1) = 1.1$, $p = .293$; see Figure S1 for model predicted values).

First, we found an overall performance improvement as revealed by an RTs decrease in the post-yoga compared to the pre-yoga session in all the blocks (see Table S12 x for post-hoc results). Overall, as expected RTs were greater for incongruent compared to congruent trials (i.e., congruency effect) in both blocks in both the pre-yoga and the post-yoga.

In terms of block-by-block performance, we found a speeding up of RTs from the non~~t~~ predictive to the predictive block only in the pre-yoga (congruent(non~~t~~ predictive - predictive): 0.05, SE = 0.01, $z_{(Inf)}$ = 4.6, p <.001; incongruent(non~~t~~ predictive - predictive): 0.04~~5~~, SE = 0.02~~1~~, $z_{(Inf)}$ = 2.9, p <.025). However, this modulation did not result in a modulation of the congruency effect between the blocks (congruency effect(non~~t~~ predictive -predictive): -0.01, SE = 0.02~~1~~, $z_{(Inf)}$ = -0.7, p =.511). In the post-yoga session, we found no effect of block in (congruent(non~~t~~ predictive - predictive): 0.004~~3~~, SE = 0.01, $z_{(Inf)}$ = 0.3, p =1.000; incongruent(non~~t~~ predictive - predictive): -0.001, SE = 0.02~~1~~, $z_{(Inf)}$ = -0.1, p =1.000). Consequently, we found no modulation of the congruency effect between the blocks (congruency effect(non~~t~~ predictive -predictive): -0.01, SE = 0.02~~1~~, $z_{(Inf)}$ = -0.4 p =.727).

	contrast	estimate	SE	df	z	p
congruent B1	pre-yoga vs post-yoga	0.15	0.01	Inf	15.4	<.001
congruent B2	pre-yoga vs post-yoga	0.10	0.01	Inf	10.9	<.001
incongruent B1	pre-yoga vs post-yoga	0.16	0.01	Inf	16.5	<.001
incongruent B2	pre-yoga vs post-yoga	0.12	0.02	Inf	7.4	<.001
pre-yoga	congruent B1 vs congruent B2	0.05	0.01	Inf	4.6	<.001
pre-yoga	congruent B1 vs incongruent B1	-0.13	0.01	Inf	-15.0	<.001
pre-yoga	congruent B2 vs incongruent B2	-0.14	0.01	Inf	-11.6	<.001

pre-yoga	incongruent B1 vs incongruent B2	0.04	0.02	Inf	2.9	.02539
post-yoga	congruent B1 vs congruent B2	0.004	0.01	Inf	0.3	1.000
post-yoga	congruent B1 vs incongruent B1	-0.12	0.01	Inf	-13.6	<.001
post-yoga	congruent B2 vs incongruent B2	-0.12	0.01	Inf	-10.1	<.001
post-yoga	incongruent B1 vs incongruent B2	-0.001	0.02	Inf	-0.1	1.000

Table S12. Post-hoc contrasts of the condition* time interaction of the ACC_M_1 model

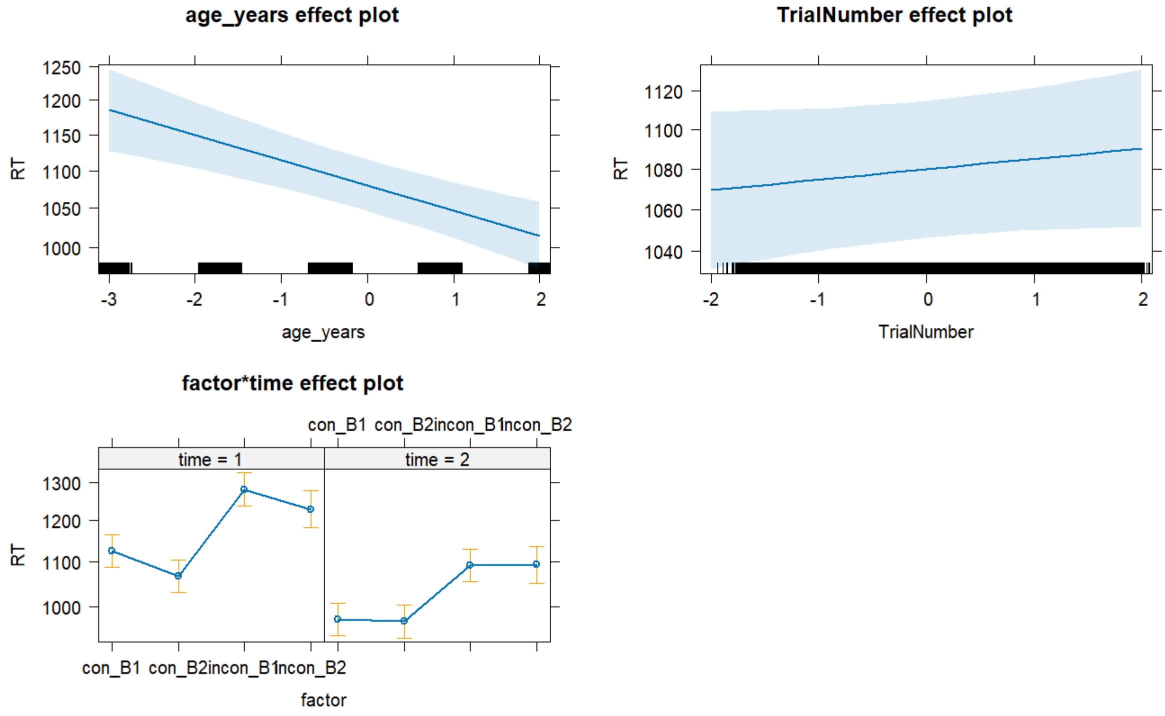

Figure S8. Results of the condition * time interaction.

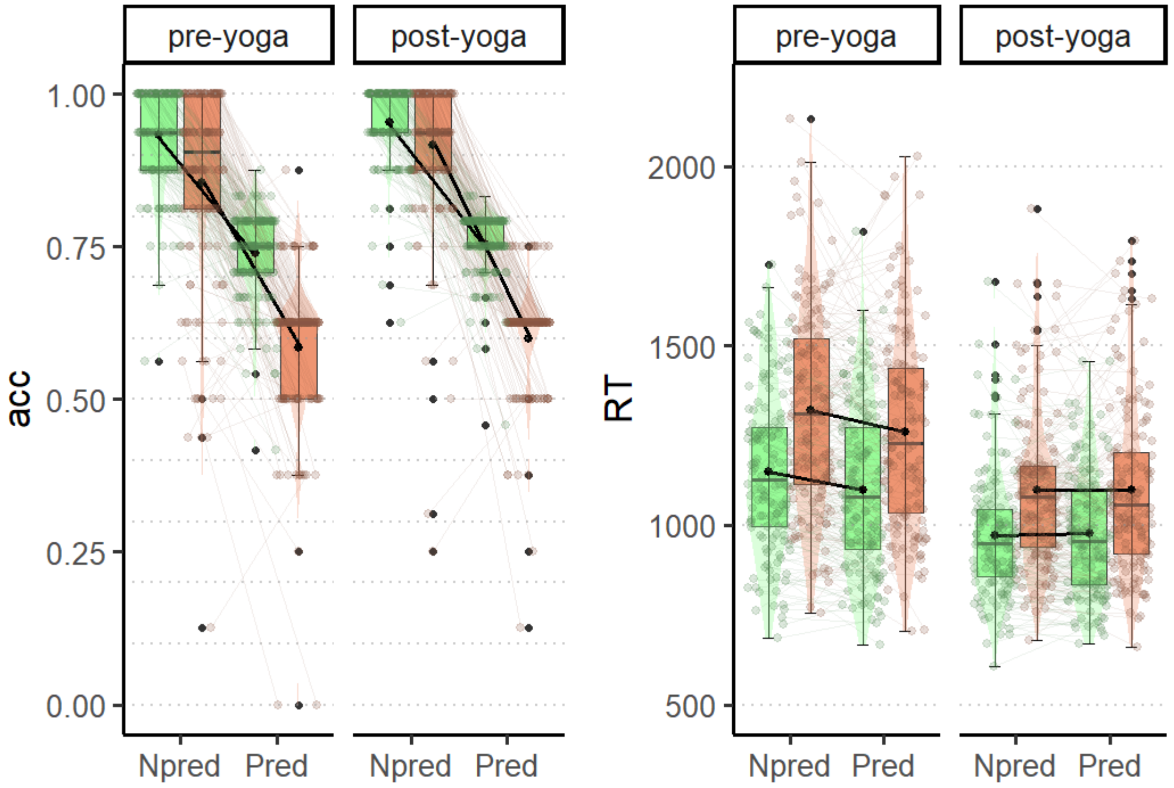

Figure S9. Accuracy and RTs in the Flanker task. Panel A. The plot displays accuracy on the y-axis, along the two blocks in the x-axis (Npred, Pred), for both congruent (green) and incongruent (orange) trials in the pre-yoga (left sub-panel) and post-yoga (right sub-panel) sessions. Panel B. The plot displays RT on the y-axis, along the two blocks in the x-axis (Npred, Pred), for both congruent (green) and incongruent (orange) trials in the pre-yoga (left sub-panel) and post-yoga (right sub-panel) sessions. In both panels, each box spans the interquartile range, with its edges indicating the first quartile and third quartile. The median is represented by a line inside the box. Additionally, individual observations are depicted as jittered points.

IES

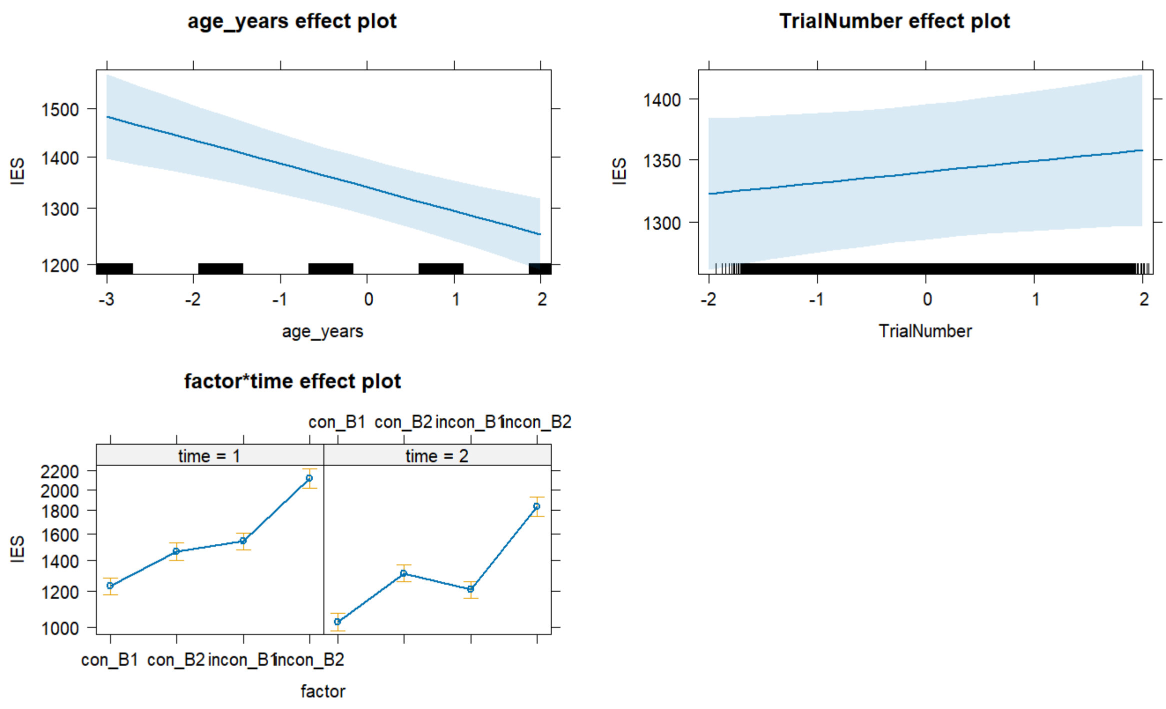

Figure S10. Results of the condition * time interaction.

Questionnaires

Variables description:

- scales → factor variable, levels: CPRS CGI total scale, PSI total scale
- time → factor variable, levels: pre-yoga, post-yoga
- score→ numeric continuous variable

The following GLMM was fitted using a Poisson family distribution:

M_Quest: score ~ 1 + scales * time + (1|id)

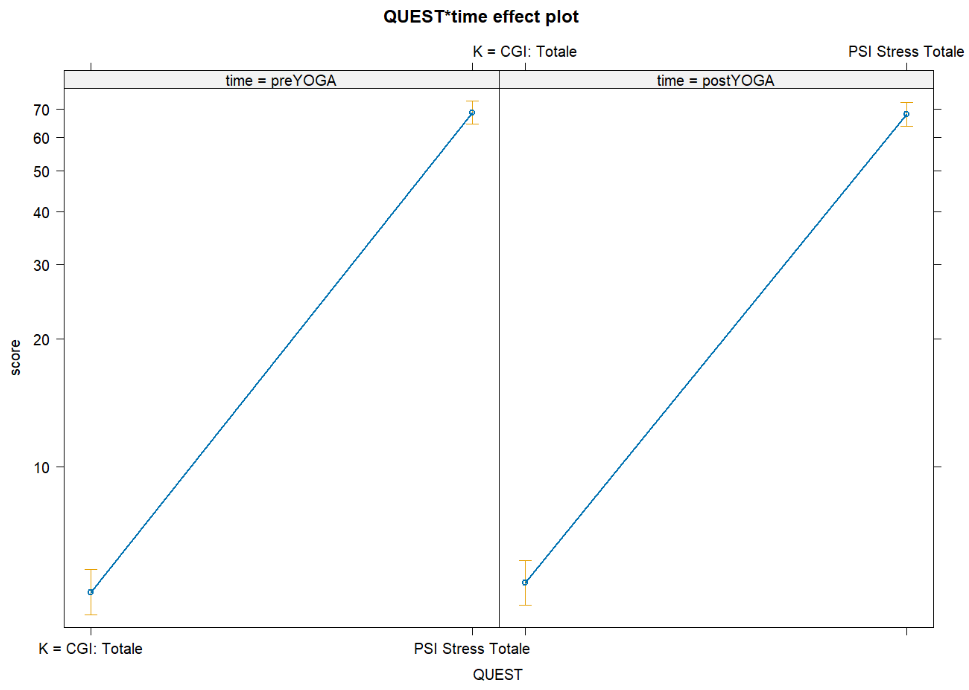

Figure S11. Results of the QUEST * time interaction.
