# Supplementary material for: Adaptive cognitive control in 4 to 7-year-old children and potential effects of school-based yoga-mindfulness interventions: an exploratory study in Italy
Source: Front Psychol. 2025 Jan 24;16:1379241. doi: 10.3389/fpsyg.2025.1379241 (PMC11803634; doi:10.3389/fpsyg.2025.1379241)
Supplement: Supplementary file 1 [file Data_Sheet_1.PDF]

Padova, December 12th, 2024

Dear Editor,

Please find enclosed a revised version of the manuscript: "*Adaptive Cognitive Control in 4 to 7-Year-Old Children and Potential Effects of School-Based Yoga-Mindfulness Interventions: An Exploratory Study in Italy*" which we submit for publication as an original research paper to *Frontiers in Psychology*.

My co-authors and I sincerely thank the Reviewers for their thoughtful and thorough evaluation of our manuscript. We are especially grateful to Reviewer 3 for accepting the manuscript and for their suggestions, which we have carefully addressed in the revised version. We also extend our thanks to Reviewer 4 for their detailed and constructive feedback, which has helped us improve the clarity and rigor of our work. Specifically, Reviewer 4 requested some theoretical clarifications regarding the concept of cognitive control adaptation and the role of conflict, as well as additional details on how our hypotheses were derived from previous literature. They also raised some points requiring minor clarifications. We have addressed all these concerns in our detailed responses and incorporated corresponding revisions into the manuscript to enhance its clarity and readability.

Additionally, during our review of the manuscript, we identified and corrected some numerical typos in the reported results. Importantly, these corrections do not alter the overall interpretation or conclusions of the study.

We have provided a detailed point-by-point response to all comments, highlighting the changes made in the manuscript (tracked). Thanks to the Reviewers' suggestions we refined and improved our manuscript, and we believe that the current version is now a stronger paper. We hope that you will find this version suitable for publication in Frontiers in Psychology.

We look forward to hearing from you again soon.

Sincerely,

Corresponding author

Reviewer 3
We sincerely thank the Reviewer for their constructive suggestions, which have been included in the revised manuscript.

**1**	**Yoga itself is a mindfulness technique. So, no need to mention Yoga – Mindfulness. Yoga is self-explanatory.**
1	We would like to thank the Reviewer for the suggestion. Although we acknowledge that yoga itself can be considered as a mindfulness technique, we would like to clarify the reason why we preferred to use the combined term "Yoga-Mindfulness" and not the two terms separately. First and foremost, not all types of yoga entail mindfulness exercises as they are currently defined. Conventional yoga methods might emphasize physical postures (asanas) or breath control (pranayama), without specifically introducing mindfulness practices. In contrast, mindfulness, as defined by Kabat-Zinn (1990, 1994), places an emphasis on methods like body scan and focused awareness, which we also included in our intervention, similarly to previous studies (e.g., Razza et al., 2015). Furthermore, although the objectives of yoga and mindfulness are similar in that they promote awareness and wellbeing, their origins and areas of emphasis are distinct.<br><br>Razza, R. A., Bergen-Cico, D., & Raymond, K. (2015). Enhancing preschoolers' self-regulation via mindful yoga. *Journal of Child and Family Studies*, *24*, 372-385.
**2**	**Is Yoga demonstrated by a qualified instructor? Please provide the certification level.**
2	We thank the Reviewer for the useful comment. The yoga activities were led by one of the authors (F.I.), who holds a Yoga Bimbi Instructor certification recognized by CSEN (National Educational Sport Center) accredited by CONI (the Italian National Olympic Committee). This certification is awarded following a specialized one-week training course focused on teaching yoga to children, culminating in an official qualification. In Italy, there are currently no additional formalized levels or nationally standardized programs specifically for becoming a children's yoga instructor.<br>The Yoga Bimbi certification provided F.I. with the necessary expertise to conduct yoga sessions tailored to the needs of children, ensuring the activities were safe, engaging, and developmentally appropriate. Furthermore, F.I. supervised another co-author, L.S., who is a trained yoga-mindfulness teacher with extensive experience but without formal certification. Together, they ensured the quality and relevance of the yoga activities in the study.<br>We have specified this information in the revised manuscript (see lines 531-535): *"The intervention was conducted by trained professionals (F.I. who holds a Yoga Bimbi Instructor certification recognized by the National Educational Sport Center [CSEN] accredited by the Italian National Olympic Committee [CONI], and L.S. who is an experienced yoga-mindfulness teacher without formal certifications), […]".*
**3**	**Page 30 – 4(Heading). Mindfulness may be termed "Pranayama and Meditation"**

3	We thank the Reviewer for this suggestion. However, as explained in our response to Comment 1, the mindfulness practices employed in this study are grounded in a specific tradition inspired by the teachings and writings of Kabat-Zinn (1990, 1994). For this reason, we believe the term “mindfulness” is the most appropriate and accurate descriptor for the methodologies used.<br>In contrast, “pranayama” and “meditation” refer to distinct and specialized practices within broader yoga traditions, which were not specifically trained or employed in this study. As such, we feel it is important to retain the term “mindfulness” to accurately reflect the practices and theoretical framework underlying our intervention.

Reviewer 4

We sincerely thank the Reviewer for their thoughtful and detailed evaluation of our manuscript. We greatly appreciate the constructive feedback which has guided us in refining and strengthening our work. In response to the Reviewer’s comments, we have made revisions to improve the manuscript’s clarity, coherence, and alignment with existing literature.

A key point raised by the Reviewer concerned the concept of adaptation. In the revised manuscript, we have provided a more comprehensive description of this concept, drawing on relevant literature to contextualize our findings. Additionally, we have clarified our hypotheses and expectations regarding reaction times (RTs) and accuracy.

We are confident that these revisions have significantly improved both the readability and the conceptual depth of the manuscript. Below, we provide detailed, point-by-point responses to each of the Reviewer's specific comments.

**1**	**The scope of the research is too large. The question of adaptive control in 4- to 7-year-olds alone is a complex topic. Combining it with a mindfulness intervention makes the ms read like two separate studies in one ms. Did the authors consider just reporting the pre-intervention measures as the first of two manuscripts with pre-post or change data forming the second ms?**
1	We thank the Reviewer for this thoughtful and constructive suggestion. We acknowledge that the manuscript is complex as scope of our study was ambitious and involved extensive resources to be conducted: organizing pre-intervention testing for children within schools, integrating the yoga-mindfulness program in the school curricula, and then conducting post-intervention testing. However, we decided not to split the manuscript into two separate papers for two main reasons. First, our primary focus was on examining the effect of the mindfulness intervention on adaptive cognitive control. While existing literature has explored developmental trajectories in similar age ranges using the DTP task (Del Popolo Cristaldi et al., 2023) and the list-wide proportion congruency Flanker task (Gonthier et al., 2021), our study's primary innovation lies in investigating the impact of a yoga-mindfulness program on adaptive CC. This focus guided the

	development of our specific hypotheses and the design of our research.<br>Secondly, we wanted to provide scientific evidence that conducting such an intervention was feasible in the context of the Italian school system.<br>For these reasons, we felt it was more scientifically appropriate to present the study as a single cohesive manuscript, emphasizing the novelty of the intervention's effects. We hope this clarifies our decision and the rationale for keeping the study's findings consolidated.
**2a**	**Adaptation is a fascinating but complex topic, especially when investigated developmentally. Couple of points of contrast between the focus of the current study and other studies reported in the literature. First, a large body of work in adaptive aspects of control have focussed on conflict as the driving signal for adaptive processes. This includes the work of Braver, Kerns, and Botvinick, which have been cited, as well as other developmentally-oriented studies by Waxer & Morton, Wilk & Morton, and others which have not been cited. In the current study, the task in Figure 1 has varying levels of predictability but no conflict. The flanker task on the other hand does have conflict, at least on incongruent trials. Is predictablity sufficient in its own right to engage adaptation or is conflict necessary? Would be good to highlight the similarities and differences between the current study and its methods and those that have been reported elsewhere.**
2a	We thank the Reviewer for these insightful comments and for the opportunity to clarify our rationale regarding the interplay between proactive and reactive control, predictability, and conflict in our study.<br><br>We acknowledge that the majority of studies investigating adaptations of cognitive control have focused on conflict as the driving signal for adaptive processes. As the Reviewer discussed, seminal work by Braver, Kerns, Botvinick, and others has highlighted how conflict, particularly in tasks such as the Flanker, Stroop and AX-CPT, acts as a key trigger for shifts between reactive and proactive control modalities. Similarly, **developmentally-oriented studies, including those by Waxer & Morton (2011) and Wilk & Morton (2012)**, which we have **cited in the revised manuscript** (see lines 93-101), have focused on how children adapt to conflict trial-by-trial (e.g., congruency sequence effects). We now added in the manuscript the following paragraph<br>*"Interestingly, behavioral and electrophysiological responses are modulated by the presence of conflict in preceding trials in adults and adolescents, but not children, suggesting greater reliance on reactive compared to proactive CC (Waxer & Morton, 2011). Additionally, children exhibit reduced engagement of neural areas, such as the anterior cingulate cortex and lateral prefrontal cortex, which are critical for moment-to-moment conflict management and adaptation (Wilk & Morton, 2012). More recent studies focusing on CC adaptation at the list or block level, provided evidence of a progressive transition from a preferential*

	use of reactive to proactive control strategies from 5 to 10 years of age (Gonthier et al., 2019).".<br><br>This local (trial-by-trial) adaptation is the scaffolding for higher-order adaptations that can be investigated at the level of a list or block of trials using, for e.g., list-wide proportion congruency manipulations (Del Popolo Cristaldi et al., 2023). As already discussed in the manuscript, both children and adults have been shown to adapt their cognitive control performance as a function of these proportion manipulations (e.g., Braem et al., 2019; Gonthier et al., 2021).<br>Our study builds upon this framework by including tasks with differing levels of predictability, some of which do not involve explicit conflict (e.g., the Dynamic Temporal Prediction task, DTP). While the Flanker task taxes interference control and contains inherent conflict in incongruent trials as well as a predictability manipulation, the DTP task taxes motor preparation and inhibitory control and contains a predictability manipulation only. This provides an opportunity to examine adaptation in contexts where conflict is absent but cognitive demands vary based on the predictability of task parameters (Del Popolo Cristaldi et al., 2023). Crucially, **we propose that predictability of cognitive demands itself can drive adaptations in CC**. Our previous work using the DTP task (Mento and Granziol, 2020; Del Popolo Cristaldi et al., 2023) has shown that variations in temporal predictability of target appearance can elicit shifts in motor preparation and inhibitory control. This aligns with recent discussions in the literature, such as Munakata et al. (2023), which demonstrate how environmental predictability (of upcoming cognitive demands) can shape cognitive control adaptations in the absence of explicit conflict. Other lines of research further corroborate the idea that control adaptation can occur in the absence of cognitive conflict. For instance, studies focusing on response inhibition (Doekemeijer et al., 2023; Giesen & Rothermund, 2014; Van Gaal et al., 2009) show that item-specific manipulations can induce automatic adaptations of inhibitory control (Logan & Cowan, 1984; Logan, 1988; Shiffrin & Schneider, 1977). We have incorporated and expanded on these points in the revised manuscript (see lines 249-259):<br>*"Importantly, the DTP task differs from classical task measuring CC adaptation as it does not include explicit conflict. While prior research has predominantly emphasized conflict as the primary trigger for shifts between reactive and proactive control modes (e.g., Botvinick et al., 2001; Braver, 2012, Kerns et al., 2004), CC adaptations can also arise from the predictability of cognitive demands, even in the absence of conflict (Munakata et al., 2023). Other lines of research further corroborate the idea that CC adaptation can occur in the absence of cognitive conflict. For instance, studies focusing on response inhibition (Doekemeijer et al., 2023; Giesen & Rothermund, 2014; Van Gaal et al., 2009) show that item-specific manipulations can induce automatic adaptations of inhibitory control (Logan & Cowan, 1984; Logan, 1988; Shiffrin & Schneider, 1977).".*<br><br>Botvinick, M. M., Braver, T. S., Barch, D. M., Carter, C. S., & Cohen, J. D. (2001). Conflict monitoring and cognitive control. *Psychological Review*, *108*(3), 624–652. https://doi.org/10.1037/0033-295X.108.3.624

Braem, S., Bugg, J. M., Schmidt, J. R., Crump, M. J. C., Weissman, D. H., Notebaert, W., & Egner, T. (2019). Measuring Adaptive Control in Conflict Tasks. *Trends in Cognitive Sciences*, *23*(9), 769–783. https://doi.org/10.1016/j.tics.2019.07.002

Braver, T. S. (2012). The variable nature of cognitive control: A dual mechanisms framework. *Trends in Cognitive Sciences*, *16*(2), 106–113. https://doi.org/10.1016/j.tics.2011.12.010

Del Popolo Cristaldi, F., Toffoli, L., Duma, G. M., & Mento, G. (2023). Little fast, little slow, should I stay or should I go? Adapting cognitive control to local-global temporal prediction across typical development. *PLOS ONE*, *18*(2), e0281417. https://doi.org/10.1371/journal.pone.0281417

Doekemeijer, R. A., Dewulf, A., Verbruggen, F., & Boehler, C. N. (2023). Proactively Adjusting Stopping: Response Inhibition is Faster when Stopping Occurs Frequently. *Journal of Cognition*, *6*(1), 22. https://doi.org/10.5334/joc.264

Giesen, C., & Rothermund, K. (2014). You Better Stop! Binding "Stop" Tags to Irrelevant Stimulus Features. *Quarterly Journal of Experimental Psychology*, *67*(4), 809–832. https://doi.org/10.1080/17470218.2013.834372

Gonthier, C., Ambrosi, S., & Blaye, A. (2021). Learning-based before intentional cognitive control: Developmental evidence for a dissociation between implicit and explicit control. *Journal of Experimental Psychology: Learning, Memory, and Cognition*, *47*(10), 1660–1685. https://doi.org/10.1037/xlm0001005

Gonthier, C., Zira, M., Colé, P., & Blaye, A. (2019). Evidencing the developmental shift from reactive to proactive control in early childhood and its relationship to working memory. *Journal of Experimental Child Psychology*, *177*, 1–16. https://doi.org/10.1016/j.jecp.2018.07.001

Kerns, J. G., Cohen, J. D., MacDonald, A. W., Cho, R. Y., Stenger, V. A., & Carter, C. S. (2004). Anterior Cingulate Conflict Monitoring and Adjustments in Control. *Science*, *303*(5660), 1023–1026. https://doi.org/10.1126/science.1089910

Logan, G. D. (1988). Toward an instance theory of automatization. *Psychological Review*, *95*(4), 492–527. https://doi.org/10.1037/0033-295X.95.4.492

Logan, G. D., & Cowan, W. B. (1984). On the ability to inhibit thought and action: A theory of an act of control. *Psychological Review*, *91*(3), 295–327. https://doi.org/10.1037/0033-295X.91.3.295

Munakata, Y., Placido, D., & Zhuang, W. (2023). What's Next? Advances and Challenges in Understanding How Environmental Predictability Shapes the Development of Cognitive Control. *Current Directions in Psychological Science*, *32*(6), 431–438. https://doi.org/10.1177/09637214231199102

Shiffrin, R. M., & Schneider, W. (1977). Controlled and automatic human information processing: II. Perceptual learning, automatic attending and a general

<table>
<tr><td></td><td>theory. Psychological Review, 84(2), 127–190. https://doi.org/10.1037/0033-295X.84.2.127

Van Gaal, S., Ridderinkhof, K. R., Van Den Wildenberg, W. P. M., & Lamme, V. A. F. (2009). Dissociating consciousness from inhibitory control: Evidence for unconsciously triggered response inhibition in the stop-signal task. Journal of Experimental Psychology: Human Perception and Performance, 35(4), 1129–1139. https://doi.org/10.1037/a0013551

Waxer, M., & Morton, J. B. (2011). The development of future-oriented control: An electrophysiological investigation. NeuroImage, 56(3), 1648–1654. https://doi.org/10.1016/j.neuroimage.2011.02.001

Wilk, H. A., & Morton, J. B. (2012). Developmental changes in patterns of brain activity associated with moment-to-moment adjustments in control. NeuroImage, 63(1), 475–484. https://doi.org/10.1016/j.neuroimage.2012.06.069</td></tr>
<tr><td>2b</td><td>Another point of contrast. Typically, in the literature, reactive control is thought to be linked to contexts that are inherently unpredictable, such as conditions in which most trials are congruent. Under these conditions, it does not make sense to actively maintain attention guiding rules but instead react to unexpected instances of conflict when they occur. These are contexts in which responses are often accurate (control is successfully instantiated) but interference costs are larger (since managing conflict happened on the fly). By contrast, proactive control is linked to contexts that are predictable, such as conditions in which most trials are incongruent. Under these conditions, it makes sense to actively maintain attention guiding rules since conflict is likely to occur. These are contexts in which interference costs are smaller (since participants are prepared to manage conflict). The authors appear to draw the opposite links between modes of control and conditions. On page 23, for example, they predict reduced interference effects in the non-predictive compared to the predictive blocks. The authors need to clarify why their predictions about when proactive and reactive modes of control will be observed, as at face value, they seem almost the opposite of what might be predicted from the models of Braver, Botvinick and others, as well as from the empirical work of Waxer & Morton, Wilk and Morton looking at conflict adaptation in development.</td></tr>
<tr><td>2b</td><td>We also agree with the Reviewer that the relationship between proactive and reactive control and predictability needs to be carefully delineated. The literature generally associates proactive control with predictable contexts (e.g., mostly incongruent blocks) and reactive control with unpredictable contexts where conflict resolution occurs on-the-fly (e.g., Braver, 2012). However, it is important to note that the use of a reactive or proactive control modality does not depend just on whether the context is inherently predictable or not; instead, it is the type of predicted cognitive demand (e.g., high or low level of conflict in the case of Flanker’s task; slow or fast SOA in the case of DTP task). As stated</td></tr>
</table>

	by the Reviewer and by Braver (2012), mostly incongruent blocks (high conflict, difficult condition), where conflict is frequent, promote proactive control as sustained readiness to manage interference is efficient, leading to reduced interference effects. Conversely, mostly congruent blocks (low conflict, easy condition), despite being predictable, may not strongly incentivize proactive control, as the majority of trials are easy, making a more relaxed, reactive strategy sufficient. Participants in these blocks often respond quickly to congruent trials and manage conflict reactively for the rare incongruent trials, resulting in larger interference effects, as noted in prior studies (e.g., Gonthier et al., 2016).<br>Unpredictable contexts, such as non-predictive (50% congruent/incongruent) blocks, add complexity. Without a clear dominant trial type, these contexts may elicit a balance of proactive and reactive strategies or rely on individual differences. However, broader task context and transitions between blocks are critical (e.g., Gonthier et al., 2021). For example, moving from a non-predictive to a mostly-congruent block likely shifts reliance from proactive to reactive control due to the reduction in frequency of conflict trials; conversely, moving from a non-predictive to a mostly incongruent block may increase reliance on proactive control as conflict becomes more frequent. These transitions highlight the dynamic nature of control adaptation across contexts.<br>This is in line with previously observed list shifting effects (Abrahamse et al., 2013; see also Gonthier et al., 2016; Kane & Engle, 2003), showing that the difference of interference between mostly congruent and mostly incongruent blocks is smaller when the mostly incongruent block is performed before the mostly congruent block than viceversa. This pattern suggests that participants implicitly acquired cognitive control settings in the mostly-incongruent block and subsequently applied them to the mostly-congruent block.<br>For this reason, for the Flanker task in our study, we predicted a greater reliance on proactive control in the first non-predictive block compared to the second mostly-congruent block.<br>To address the Reviewer's concerns, we have better specified our expectations:<br>Lines 263-273: *"The blocks are designed to elicit greater reliance on, respectively, proactive and reactive control strategies. The non predictive block requires increased control to manage cognitive interference. Transitioning to the second predictive block (mostly congruent) introduces a higher proportion of congruent trials, creating an environment predictive of lower conflict demands. As a result, we expected greater reliance on reactive control in responding to incongruent trials within this block. This shift should result in an overall increase in cognitive interference in the predictive compared to the non predictive block. Therefore, efficient CC adaptation in this task is reflected by reduced congruency effect (speed-accuracy trade-offs) in the not predictive compared to the predictive block."*
3	**The ms includes frequent use of acronyms. Some are generally familiar to psych readers so are probably fine (e.g., SOA). However, others are hard to remember (e.g., LW manipulation) and would be better spelled out in full. See also line 1011. DTP is not an informative subtitle.**

3	We thank the Reviewer for this helpful suggestion. To improve clarity and readability, we have replaced the abbreviations ‘LW’ and ‘LWPC’ with the full terms ‘list-wide’ and ‘list-wide proportion congruency’ throughout the manuscript. Additionally, we revised the subtitles to make them more descriptive, changing ‘DTP’ to *“The Dynamic Temporal Prediction task: Inverse Efficiency Score”* and ‘Flanker’ to *“The Flanker task: Inverse Efficiency Score.”* (see lines 673 and 715). Furthermore, to ensure that the abbreviation ‘DTP’ is easier to recall, we consistently included the word *“task”* whenever the abbreviation is used.
**4**	**Some of the predictions were not immediately obvious. For example, why do the authors predict faster detection of targets at long compared to short SOAs? Wasn't predictability the foundation of adaptation according to the authors (see line 61-62? Long versus short SOAs are equally predictable -- it's just that one is longer than the other. Not clear.**
4	We thank the Reviewer for their insightful comment. The prediction that participants would detect targets faster at long compared to short SOAs is grounded in well-established findings from the literature on the "hazard function". This refers to the idea that response promptness may rely on dynamic computations of conditional probabilities, reflecting the likelihood of relevant events occurring at a specific moment, given that they have not yet occurred. Experimentally, the hazard function translates into the well known “Variable foreperiod effect” (Woodraw, 1913; Luce, 1986). This phenomenon suggests that as the interval preceding a target (i.e., the foreperiod) increases, motor preparation to respond also intensifies (Los et al., 2017; Niemi and Näätänen, 1981) as the probability of stimulus occurrence depends on the time elapsing by itself.<br>However, this increased motor readiness also necessitates greater inhibitory control to prevent anticipatory responses. It is important to emphasize that these effects occur at the *local* level - that is, they reflect immediate trial-by-trial dynamics based on the foreperiod (i.e., SOA) duration (Mento and Granziol, 2020).<br>We have better specified this in the manuscript (lines 361-367):<br>*“These two foreperiods reliably induce hazard-related, local prediction effects on reaction times (RTs) in children. Specifically, this manipulation is expected to bias subjective temporal expectancy (Luce, 1986; Karlin, 1959; Woodrow, 1914), leading to faster detection of targets at the longer SOA and slower detection at the short SOA (Niemi and Näätänen, 1981). Indeed, as the temporal interval preceding a target increases, motor preparation to respond also intensifies.”.*<br><br>In addition to these local effects, our study also examines *global* adaptation patterns driven by the distribution of foreperiods within a block. By manipulating the percentage of long versus short SOAs, we created "slow" blocks (70% long SOAs) and "fast" blocks (70% short SOAs). These global adaptations bias participants toward overall slower or faster reaction times, reflecting an

	adjustment to the speed demands of the block. We have made this explanation clearer in the revised manuscript (see lines 373-375):<br>*“Crucially, "fast blocks" are expected to elicit faster responses than "slow blocks", reflecting an adjustment to the speed demands of the block”.*
**5**	**Line 351. If children are required to respond as quickly as possible (line 346), why were they instructed to not to press too soon or too late? Should they not have simply been told not to respond too late?**
5	We appreciate the Reviewer’s observation and understand the concern.<br>**The instruction to avoid pressing too early specifically targeted anticipatory responses (i.e., responses made before the target appeared).** In the task design described in the manuscript, the target (a cartoon animal) appeared within a warning stimulus (a black camera lens surrounded by a circle). This warning stimulus could unintentionally trigger anticipatory responses, as children might mistakenly press the button upon seeing the camera lens instead of waiting for the target.<br>To address this, participants were instructed to respond as quickly as possible *only when the target appeared* and to avoid pressing too early.<br>In the manuscript (lines 350-352):<br>*“Participants were required to press the spacebar of the keyboard with the index finger of their dominant hand as quickly as possible* ***at target occurrence.*** *”.*<br>This instruction was reinforced during practice trials, where children received trial-by-trial feedback. If they made an anticipatory response (before the target appeared) or too soon after the target's onset (<150 ms), they were advised accordingly. We have clarified this in the revised manuscript by adding the following details (see lines 394-398):<br>*"During practice participants received trial-by-trial feedback based on their performance (i.e., puzzled face for wrong, anticipatory [response before target appearance] or premature [<150 ms after target appearance] responses, sleepy face for correct but delayed [> 800 ms after target appearance] responses, smiley face for correct and fast responses).".*
**6**	**It is not exactly clear what the Questionnaires were meant to measure. Why would a clinical measure of parents' perception of their children's EF be influenced by a mindfulness training intervention?**
6	We appreciate the Reviewer’s concern and would like to clarify our rationale for including the Conners Parent Rating Scale (CPRS) and the Parenting Stress Index (PSI) in our study. The decision to include these measures was based on multiple considerations.<br>First, consistency with prior research played a key role. **Almost all studies evaluating the effects of mindfulness interventions have relied solely on questionnaires to assess changes in executive functioning** (EF; i.e.,

	cognitive control) and related behaviors. For instance, the study by Crescentini et al. (2016) used the Child Behavior Checklist (CBCL), CPRS, and other self-report measures to assess the effects of mindfulness training. Their findings suggested positive outcomes in terms of attention and internalizing problems, as noted in teacher ratings. Similarly, other studies have documented improvements in stress management, reduced negative emotions and thoughts, and enhanced school performance, attention, and cognitive control (e.g., Bazzano et al., 2022; Frank et al., 2017; Good et al., 2016; Hart et al., 2022; Serwacki & Cook-Cottone, 2012; Sibinga et al., 2016).<br>In our study, **we hypothesized that the yoga-mindfulness intervention would not only improve children's cognitive control in more controlled tasks but also translate to everyday behaviors. This in turn should also be reflected in reduced parental stress.** Mindfulness-based interventions are thought to improve self-regulation and stress management, which would naturally extend to the daily lives of children and, consequently, their parents. We therefore anticipated that parents' perceptions of their children's behavior, as well as their own stress levels, would improve following the intervention.<br>We have clarified this point in the revised manuscript by emphasizing it when describing our hypotheses (see lines 285-294):<br>*"Finally, we evaluated the impact of the yoga-mindfulness intervention on children's daily behaviors and parental stress through parental questionnaires, specifically the Conners' Parent Rating Scales - Revised (CPRS-R; Conners, 2001; Conners et al., 1998; Italian adaptation by Nobile et al., 2007) and the Parenting Stress Index (PSI; Abidin, 1995; Italian adaptation by Guarino et al., 2008). Consistent with previous research (Crescentini et al., 2016), we anticipated a general reduction in behavioral problems and parental stress as a result of the intervention (H3). Indeed, mindfulness-based interventions have been shown to improve cognitive control, attention and stress management (Bazzano et al., 2022; Frank et al., 2017; Good et al., 2016; Hart et al., 2022; Serwacki & Cook-Cottone, 2012; Sibinga et al., 2016).".*
**7**	**Table 1, line 1070. There is missing information in the Table somewhere. Why, for example, are the parameter estimates and the SE for Slow-1 and Fast-1 both equal 0.06 and 0.01 but the z for Slow-1 is 5.2 but for Fast-1 is 8.1? Hard to understand.**
7	We thank the Reviewer for bringing this to our attention. Upon reviewing our analysis (also available on OSF), we identified some errors in the reporting of our results. These numerical inaccuracies have been corrected in the revised manuscript (see lines 674-677, Table 1, Table 2 and Table 4); however, the overall findings and conclusions of the study remain unchanged.<br>To improve clarity, we have also provided more detailed explanation in Table 1 and Table 3 captions:<br>*"The Table presents post-hoc contrasts of the block × time interaction, comparing the pre-yoga and post-yoga conditions across multiple contrasts. For each contrast, the table includes the contrast estimate, standard error (SE), degrees of*

	<u>*freedom (df), z-statistic, and p-value. These contrasts help to highlight the impact of the yoga intervention on adaptive cognitive control, and the statistical significance is indicated for each contrast (with p-values < .05 for most of the comparisons).”*</u>*.*
**8**	**Avoid use of evaluative terminology when discussion adaptation. See line 1099. Adaptation of control is not inherently an improvement -- it's simply a change in the manner in which control is deployed. Some environments call for reactive control and that makes sense. Other environments allow for proactive control. One is not better than the other, nor does the change denote improvement -- it denotes adaptation.**
8	We thank the Reviewer for this insightful comment. At the specified line (1099 of the original manuscript, 678 of the revised manuscript), the term *improvement* was used to describe the overall performance across all blocks, as evidenced by a decrease in total IES in the post- compared to the pre-yoga session. This reflects a general enhancement in task performance rather than a specific improvement in adaptation. We have made this clearer in the revised manuscript (see lines 678-680 and 728):<br>*“First, we found an overall performance improvement as revealed by a decrease in IES in the post-yoga compared to the pre-yoga session, in all the blocks except for the third block (see Table 1 for post-hoc contrasts).* <u>*Regarding specifically adaptation*</u>*, as expected (H1a), only late adaptation occurred [...]”.*<br><br>The Reviewer is absolutely correct that cognitive control adaptation refers to changes in the deployment of reactive or proactive control based on contextual demands. Adaptation, by itself, is not inherently an improvement - it reflects a dynamic adjustment to the task environment. Reactive control may be optimal in one context, while proactive control may be better suited to another; neither mode is intrinsically superior.<br>Importantly, one of the aims of this study was to examine whether a yoga-mindfulness training session could influence the degree of this adaptive flexibility. When we refer to “optimized/improved adaptation” (e.g., line 689), we are specifically comparing the two testing sessions, where changes in adaptation were evaluated in terms of speed-accuracy trade-offs (i.e., Inverse Efficiency Score).<br>To address the Reviewer’s concern, we have replaced evaluative terms such as *improvement* with more neutral phrasing (e.g., increased or decreased degree of adaptation, e.g., see lines 689, 852) to accurately reflect the observed changes between the two timepoints without implying value judgment. We hope these revisions better align with the Reviewer’s expectations.
**9**	**Typos/missing words. See line 684: "...the null model the Akaike...".**
9	We thank the Reviewer for pointing out the typo. We have corrected the sentence

	to read: *"[...] the null model* <u>*using*</u> *the Akaike Information Criterion [...]"*. Additionally, we have carefully reviewed the manuscript to identify and correct any other typographical errors.
